# Identification and Structural Characterization of Twisted Atomically Thin Bilayer Materials by Deep Learning

**DOI:** 10.1021/acs.nanolett.3c04815

**Published:** 2024-02-26

**Authors:** Haitao Yang, Ruiqi Hu, Heng Wu, Xiaolong He, Yan Zhou, Yizhe Xue, Kexin He, Wenshuai Hu, Haosen Chen, Mingming Gong, Xin Zhang, Ping-Heng Tan, Eduardo R. Hernández, Yong Xie

**Affiliations:** † Key Laboratory of Wide Band-Gap Semiconductor Technology & Shaanxi Key Laboratory of High-Orbits-Electron Materials and Protection Technology for Aerospace, School of Advanced Materials and Nanotechnology, Xidian University, Xi’an 710071, China; ‡ Department of Materials Science and Engineering, University of Delaware, Newark, Delaware 19716, United States; § State Key Laboratory of Superlattices and Microstructures, Institute of Semiconductors, Chinese Academy of Sciences, Beijing 100083, China; ∥ Phonon Engineering Research Center of Jiangsu Province, School of Physics and Technology, Nanjing Normal University, Nanjing 210023, China; ⊥ School of Materials Science and Engineering, Northwestern Polytechnical University, Xi’an 710072, China; # Instituto de Ciencia de Materiales de Madrid (ICMM-CSIC), 28049 Madrid, Spain

**Keywords:** Twist angles, Transition metal dichalcogenides (TMDs), Deep learning, OpenCV, Raman

## Abstract

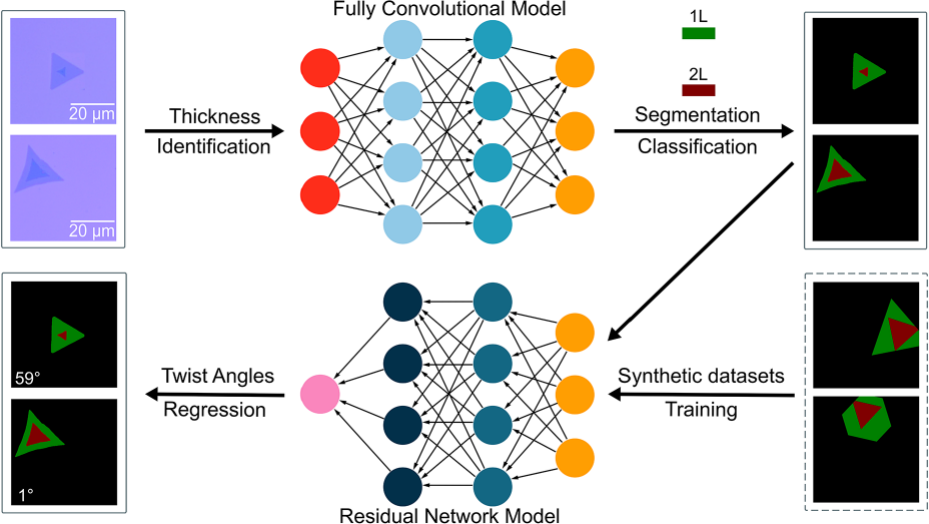

Two-dimensional materials are expected to play an important role in next-generation electronics and optoelectronic devices. Recently, twisted bilayer graphene and transition metal dichalcogenides have attracted significant attention due to their unique physical properties and potential applications. In this study, we describe the use of optical microscopy to collect the color space of chemical vapor deposition (CVD) of molybdenum disulfide (MoS_2_) and the application of a semantic segmentation convolutional neural network (CNN) to accurately and rapidly identify thicknesses of MoS_2_ flakes. A second CNN model is trained to provide precise predictions on the twist angle of CVD-grown bilayer flakes. This model harnessed a data set comprising over 10,000 synthetic images, encompassing geometries spanning from hexagonal to triangular shapes. Subsequent validation of the deep learning predictions on twist angles was executed through the second harmonic generation and Raman spectroscopy. Our results introduce a scalable methodology for automated inspection of twisted atomically thin CVD-grown bilayers.

Inspired by magic-angle graphene,^1,2^ twisted bilayer graphene and transition metal dichalcogenides (TMDs) have emerged as a promising platform for the study of Moiré physics, encompassing a range of phenomena such as Hubbard physics,^3,4^ superconductivity,^5^ or valley polarization.^6^ The twist angle in TMD bilayers can significantly alter their correlated electronic phases and their optical properties.^7^ For example, in the twist angle (2° ≤ θ < 6°), low-frequency interlayer shear and layer breathing modes exhibit rapid change with the twist angle θ.^8^ Additionally, the formation of the moiré Brillouin zone introduces new energy subbands in twisted MoS_2_ bilayers with twist angles close to 0° or 60°^9^ or high-lying excitons in bilayer WSe_2_, which can be tuned over 235 meV by enforcing different twist angles in the range 0° to 60°.^10^ The electric field control of the 2H bilayer MoS_2_ interlayer exciton at room temperature is possible due to the out-of-plane electric dipole.^11^ Chemical vapor deposition (CVD) can be used to fabricate bilayer graphene^12^ and bilayer TMDs with different twist angles,^13−17^ i.e., different stacking arrangements between the two layers. Typically, only 0° (AA stacking, or 3R) or 60° (AB stacking, or 2H) arrangements are possible for the second layer on bilayer TMDs, as these are energetically more favorable.^16,18^ Reflectivity spectra have shown an A and B exciton energy difference of 49 meV between the 2H and 3R bilayer MoS_2_.^15^ Reverse-flow chemical vapor epitaxy provided a way to controllably grow high-quality bilayer TMD single crystals with different growth temperatures.^16^

Second harmonic generation (SHG) spectroscopy is frequently employed to characterize the twist angles of TMDs.^19,20^ This reliable method determines the orientation of exfoliated flakes and subsequently enables the stacking of homo- or heterolayers using a dry transfer technique.^21−23^ In addition to SHG, differential reflectance spectroscopy can also be used for characterizing TMDs, specifically by identifying twist angles through the transition of interlayer excitons.^15,24^ Raman spectroscopy, particularly low wavenumber Raman spectroscopy,^25,26^ is another common method used for this purpose.^8,16,27−29^ For cases where atomic precision is required, transmission electron microscopy (TEM) can be employed to determine the twist angles of graphene.^30^ Further augmenting the atomic precision of characterization techniques, scanning tunneling microscopy (STM) offers local probing of twist angles with atomic level resolution.^31^ Although accurate and reliable, these experimental techniques are costly, require specialized equipment, and are time consuming. It is therefore desirable to develop alternative structural characterization techniques that are cost-effective, fast, and easily implemented without compromising accuracy and reliability.

Although the thickness of two-dimensional (2D) materials is normally confirmed by atomic force microscopy (AFM), Raman, etc.,^32^ optical contrast is frequently adopted by experienced researchers due to its speed and simplicity.^33^ Recent developments in artificial intelligence (AI) have led to the adoption of new techniques for processing microscopy image data sets of layer thicknesses, edges, dimensions, etc.^34−36^ For example, an autonomous robotic search and stacking of graphene flakes was proposed to detect up to 400 monolayer graphene samples in 1 h.^34,35^ Unsupervised Machine Learning (ML) and Deep Learning (DL) techniques have been used to classify 2D materials into different categories.^37−40^ In combination with an automatic optical microscope stage, the desired 2D materials can be searched automatically.^39^ However, to the best of our knowledge, no automatic procedure for determining the twist angle in bilayer atomically thin materials (e.g., TMDs and graphene) has been described up to now. Presumably, the DL model may lack sufficient accuracy due to the absence of adequate experimental data for training.

In this work, a systematic methodology is reported that demonstrates the potential of DL and image processing tools for determining the twist angles in CVD-grown bilayer TMDs and graphene. Specifically, we trained four different DL algorithms to identify the thickness of the CVD-grown flakes. The twist angle in individual bilayer TMD flakes can be estimated by means of image processing tools, such as implemented in OpenCV.^41^ This procedure, illustrated below, is nevertheless slow and not effective for large-scale sample analysis. To circumvent this problem, we developed a second DL model to characterize the twist angle of individual flakes in an efficient way. All codes and data sets are open access and freely available, provided with user-friendly instructions. Our work aims to provide new tools designed to facilitate and make more effective structural characterizations of CVD-grown twisted TMD samples, with extended applicability to CVD-grown graphene and hexagonal boron nitride (h-BN) and other CVD-grown 2D materials.

In our study, optical micrographs of CVD-grown bilayer atomically thin materials are captured as shown in Figure 1 (for MoS_2_) and supplementary for graphene (Figure S15).^12^ These images are then processed and utilized to train a convolutional neural network (CNN) for the identification of flake thickness. Subsequently, a different CNN model, developed using a synthetic data set, is used to predict the twist angles of the flakes, with these predictions displayed on the corresponding images. The workflow diagram of the process to identify the twist bilayer of TMDs is visually shown in Supporting Information Figure S1.

**Figure 1:**
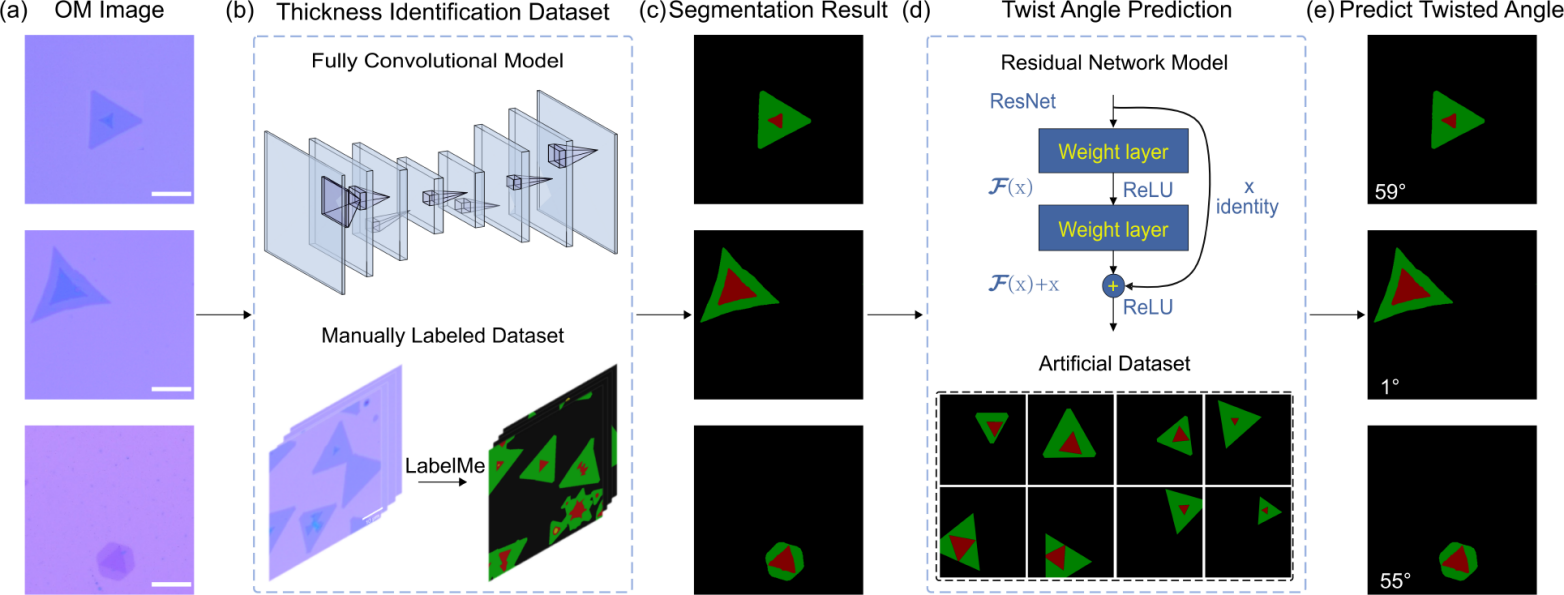
Identification and analysis of optical micrographs of CVD-grown bilayer atomically thin materials (e.g., TMDs) using deep learning. Images of the TMDs are shown as an example. (a) Original optical micrographs (OMs) captured by an optical microscope. (b) Images processed and labeled using LabelMe, followed by training via a convolutional neural network (CNN) employing various classification methods. (c) Typical outcomes of the TMD thickness derived from the processing in step (b). (d) The regression CNN model trained by the artificially generated data set for twist-angle prediction. (e) Twist angles predicted by using the CNN model from (d) are shown at the left corner at each image. Scale bar: 10 μm.

We utilized deep learning techniques to determine the thickness of atomically thin flakes, harnessing a supervised neural network trained on manually labeled images. These atomically thin flakes were initially distinguished by optical contrast and subsequently verified through AFM and Raman spectroscopy,^13,14^ as detailed in Supporting Information Section 1.1.

Figure 2 showcases the adeptness of our segmentation DL models in classifying flakes into monolayer (1L), bilayers (2L), and thicker layers (TL) with remarkable precision, as further elaborated in Figure 2 and Figure S7. Figure 2(a) shows an original unprocessed microscopy image. Figures 2(b–e) illustrates the manual labeling of background, monolayer, bilayer, and thick layers.

**Figure 2:**
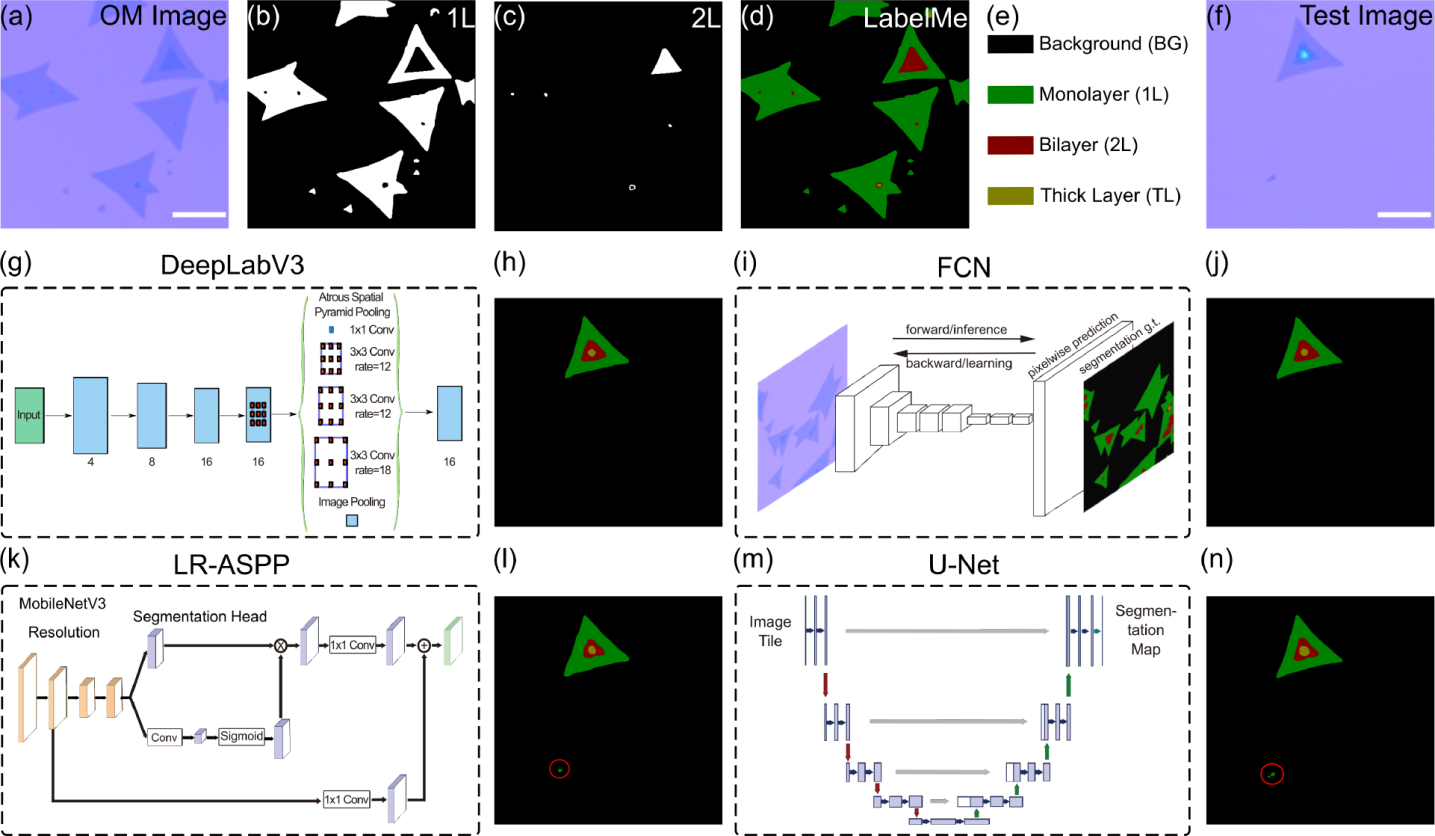
Segmentation techniques for classifying thickness in atomically thin CVD-grown bilayer flakes. (a) Optical micrographs of a bilayer MoS_2_ flake. (b,c) Detail of the labeling for single and bilayer regions. (d) Final labeled result identifying thickness variations. (e) Explains the color labels, correlating colors to specific thickness levels. (f) Optical micrographs of another bilayer flake used as a test image. (g–n) Four CNN models and segmentation results employing DeepLabV3, FCN, LR-ASPP, and U-Net models, respectively. Notably, the U-Net model excels in recognizing the contours of imperfect triangular flakes. Scale bar: 10 μm.

In our evaluation, four DL models were rigorously tested: DeepLabV3,^42^ Fully Convolutional Network (FCN),^43^ MobileNetV3 (LR-ASPP),^44^ and U-Net,^45^ each employing distinct segmentation strategies and architectures. For instance, DeepLabV3 integrates a backbone architecture for feature extraction with advanced techniques, such as dilated convolutions and spatial pyramid pooling. The FCN model, tailored for semantic segmentation, ensures that the output size matches the input. Meanwhile, LR-ASPP, a variant of DeepLabV3′s ASPP, aims for efficiency in mobile and edge computing. Notably, U-Net, recognized for its effectiveness in biomedical image segmentation, features a distinctive U-shaped architecture. Our implementation utilized Python 3.8 and Pytorch 1.10,^46^ with U-Net emerging as the most adept in capturing the nuances of shape, particularly evident in cases of distorted triangular overlays. Compared to the original micrographs, U-Net’s segmentation reveals enhanced detail in both monolayer and bilayer configurations (Figure 2(n)), affirming its selection as the primary model for our study. For a comprehensive understanding of the labeling process and additional model comparisons, readers can refer to the Supporting Information (Section 2.1).

Once the pixels in an experimental image have been classified, either by a human or by any of the trained DL models described in Sec. I of SI, and the resulting flakes color-coded according to their thickness, it is possible to determine the twist angle formed between an underlying single-layer and an overlying second layer using appropriate image analysis software, such as OpenCV.^41^ It contains useful functionalities to e.g. determine contour lines enclosing a particular flake and measure the corresponding enclosed area, fit an approximate polygonal shape, or find the minimum desired polygonal shape that encloses a given set of pixels in the image. Given that TMDs typically exhibit a triangular shape, we employ triangles as the chosen polygonal shape, leading to an estimation of the corner positions. By carrying out this process for each of the layers in a flake, it is possible to estimate the twist angles between each pair of layers from their corner positions by simple trigonometric calculation.

Nevertheless, the use of image analysis software as described above has several disadvantages. First, it is not easily automated, requiring human intervention and thus resulting in a slow and tedious process that cannot be applied efficiently on a large scale. A better alternative is to train a DL model to directly predict the twist angles from the appropriately cropped experimental image of a twisted bilayer [see Figure 1(d,e)]. Training such a model would require a large database of preprocessed experimental images, for which the twist angles had been previously determined. However, there is a more practical and expedient procedure, which consists of employing a synthetically generated image database. As can be seen in Figure 1 and Figure 2, experimental samples of MoS_2_ bilayer flakes typically consist of two roughly triangular shapes, with the rotation angles in the range of 0 to 60°. It is trivial to generate large numbers of synthetic images of rotated pairs of superimposed triangles. This adds the advantage that such images can be made to sample homogeneously all possible twist angles, while training on labeled experimental images would result in a bias toward the experimentally observed twist angles. It is even possible to generate synthetic images that mimic more faithfully the experimental ones, by, e.g., cropping corners of the triangles or introducing random noise to their edges.

In the process of generating the data sets for training the second neural network, a sequence of images is created, including double-layered polygons that morph sequentially from hexagons to truncated triangles and finally into triangles (as illustrated in Figure 3(a)). Formation of the outer polygon begins with the careful selection of random variables such as central coordinates, side lengths, and rotational angles, promoting visual diversity. Once the outer polygon is defined, an inner polygon is positioned within it. The inner polygon’s location, rotation, and side length are determined through random selection, while ensuring its side length is equal to or less than the outer polygon’s, preserving their hierarchical nesting and maintaining distinct spatial separation between them.

**Figure 3:**
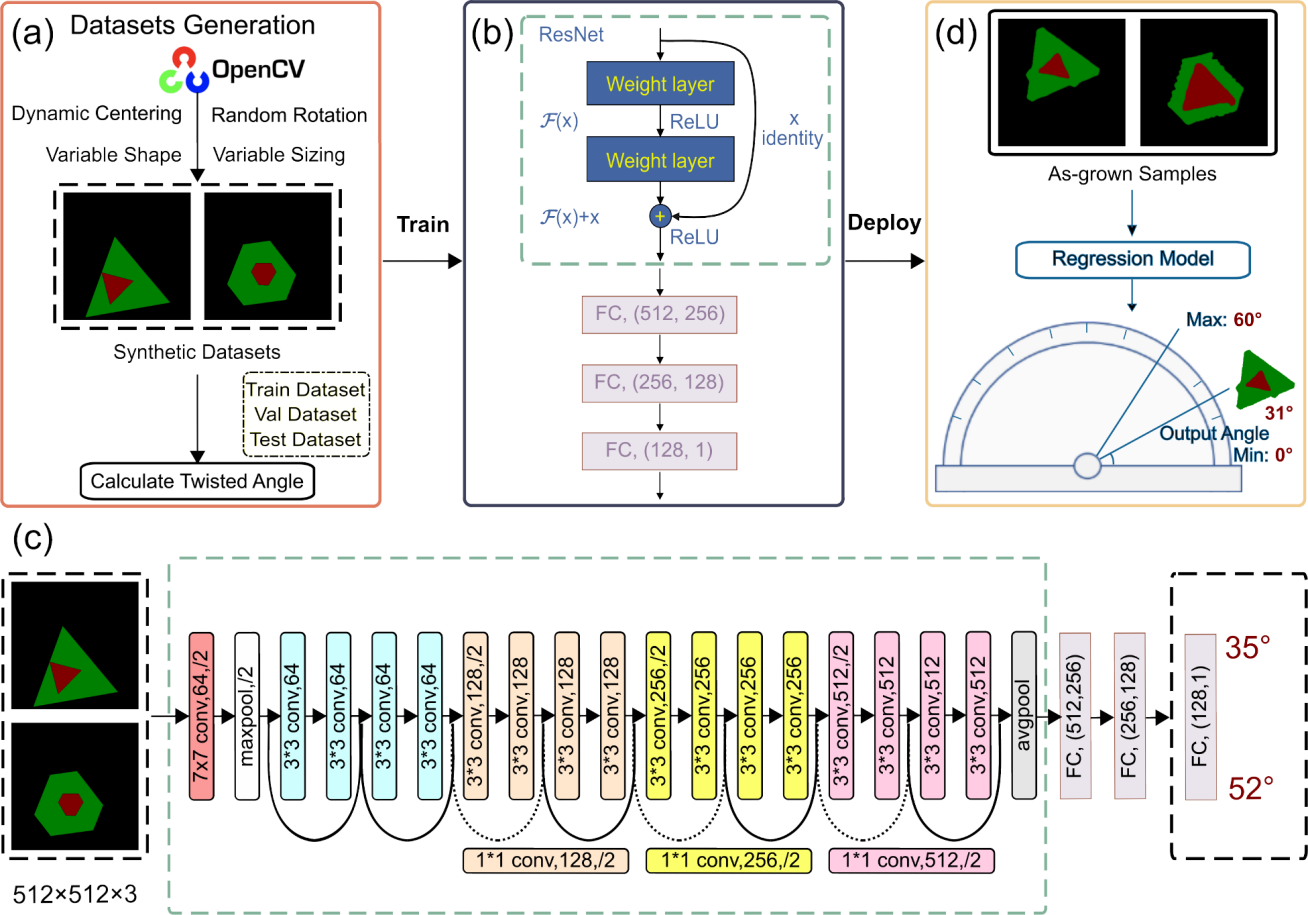
Deep learning approach for recognizing twist angles in atomically thin bilayer flakes. (a) Synthetic data sets illustrating varying twist angles in uniformly colored MoS_2_ flakes postsegmentation. (b) ResNet CNN model training using the linear regression approach on the data sets from (a). (c) The detailed structure of ResNet with input training synthetic data sets and output twist angles of the data sets. (d) Prediction of twist angles for actual as-grown MoS_2_ bilayer samples using the trained CNN model.

The angle formed by a vertex of each polygon and its respective center is calculated, providing a measure to assess the angular difference between them. This angular variation is then incorporated into the filename of the stored image, serving as a perceptible geometric attribute label. Consequently, the generated data sets, enriched with clear geometric annotations and offering a wide variety of forms, become a valuable resource for training data sets in the subsequent recognition of twist angles in CVD-grown samples.

After that, a *Residual Network* (ResNet)^47^ Convolutional Neural Network (see Figure 3) is trained to predict the twist angle by regression to a synthetically generated database of more than 10,000 images. The input images are RGB images with a resolution of 512 × 512 pixels obtained from Figure 3(a). In model design, the choice between using a single-layer or multilayer approach for regression tasks hinges on the complexity of the data relationship. A direct 512 to 1 dimension reduction in the network might be suitable for a simpler, linear problem, while a more gradual decrease, such as 512–256–128–1, tends to perform better for capturing complex, nonlinear relationships in the data (as shown in Figure 3(b) and (c)). In the latter approach, additional layers aid the model in learning nuanced data patterns, offering a potential boost in the predictive accuracy for intricate problems. Upon being input into the ResNet network, the final angles are obtained, as illustrated in Figure 3(b) and (c). After training the CNN regression model, the real as-grown bilayer MoS_2_ micrographs after the first deep learning model are used as input, and the twist angle of the bilayer MoS_2_ is obtained through this second neural network. The capabilities of the model are illustrated in Figure 4. The identification of twist bilayer MoS_2_ using OpenCV is also demonstrated in Figure S8 and shown in Figure 4 as a comparison for the DL methodology. It can be concluded that the CNN regression model we used here can identify the twist angle in bilayer MoS_2_ while being tolerant of shape irregularities. For bilayer graphene synthesized through CVD, it is evident that different edges can correspond to varying twist angles in the secondary layer^12^ (Figure S15). Demonstrably, our methodology proficiently enables the identification of such twist angles within hexagonally shaped, CVD-grown bilayer graphene, offering insightful exploration into its structural intricacies, as depicted in Figure S15.

**Figure 4:**
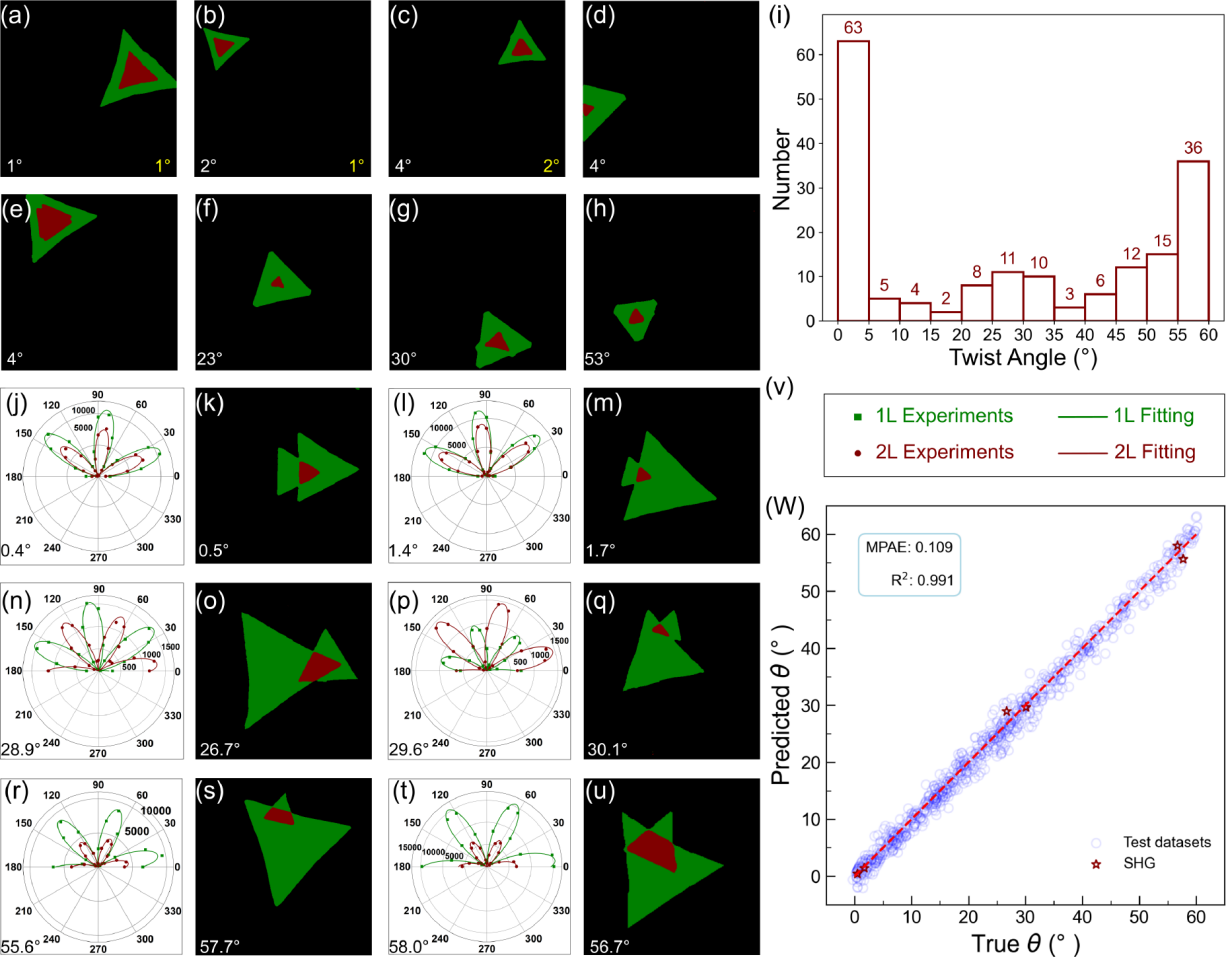
Performance evaluation of the twisted bilayer MoS_2_ identification by the CNN model. (a)–(h) Predicted angles for different flakes, showcasing the efficacy of the CNN-based model (indicated in the bottom-left corner of each subfigure) in comparison to angles obtained via OpenCV (displayed in the bottom-right corner). It is noteworthy that in numerous instances OpenCV was unable to identify the angles, resulting in absent data in the bottom-right corner of some subfigures. (i) Histogram of the quantity of bilayer MoS_2_ at various twist angles. (j)–(u) Second harmonic generation (SHG) and thickness identification for the corresponding samples with (v) serving as the legend. (w) Comparison between true and predicted twist angles θ using the artificially generated test data sets and SHG data set in (j)–(u), respectively.

To verify the identification of the twist angles of CVD-grown bilayer MoS_2_, SHG measurements are performed on these samples with various twist angles as shown in Figure 4(j)–(v). The true and predicted twist angle θ using the artificially generated test data sets and SHG data set is summarized in Figure 4(w), showing the reasonable accuracy of our model.

One possible application of our methodology could be to select more samples for probing their optical property correlations. The strain, defects, and doping of the inhomogeneity of the individual bilayer CVD-grown flakes could influence the Raman spectra, making the interpretation of Raman signal challenging.^48−54^Figure 5(a) shows the Raman spectra of the CVD-grown bilayer MoS_2_ samples with twist angles ranging from 0 to 60°. The three vertical dashed lines correspond to the peak positions of the second-order Raman modes *E*′(M)^LO_2_^-LA(M) (∼150 cm^–1^), *A*_1_^′^(M)-LA(M) (∼178 cm^–1^), and TA(K) (∼190 cm^–1^).^55^ In addition, several branches of Moiré phonons are observed in the Raman spectra. Moiré phonons refer to zone-center phonons in twisted bilayer MoS_2_, which are folded from the off-center phonons in monolayer MoS_2_ due to the periodic Moiré potential and thus exhibit a shift in peak frequency with the change of twist angle.^28^ The arrows indicate the assigned Moiré phonon modes, including folded TA (FTA), folded LA (FLA,) and folded *A*_1_^′^ (F*A*_1_^′^) modes, which exhibit high-frequency sensitivity to the twist angle, similar to the previous results.^28^Figure 5(b),(c) summarizes the experimental and calculated frequencies of the three Moiré phonon modes for comparison. The experimental peak positions of Moiré phonons agree well with the theoretical ones^28^ based on the twist angle of twisted bilayer MoS_2_ and the phonon dispersion of monolayer MoS_2_. Note that the frequencies of the F*A*_1_′ mode on the CVD-grown bilayer MoS_2_ diverging relatively from the theoretical curve in the range of 24.6° to 34.7° may arise for different reasons, where strain is very likely as *E*′ and *A*_1_′ modes also show behaviors of strain-induced shifts.^51^ We did not discuss the results of the twist angles at 0–5° and 55–60° in detail because the case is very complicated due to the phonon renormalization induced by lattice relaxation.^8^

**Figure 5:**
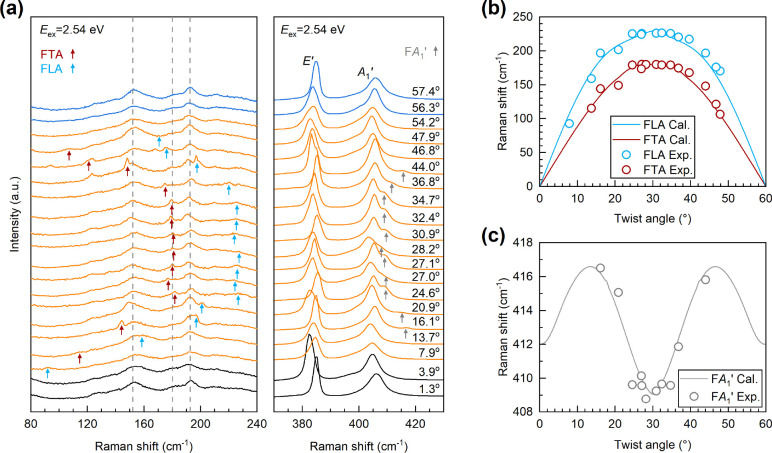
Moiré phonons in twisted CVD-grown bilayer MoS_2_. (a) Raman spectra of CVD-grown bilayer MoS_2_ with the excitation energy (*E*_ex_) of 2.54 eV. Peaks assigned to Moiré phonons modes^28^ including FTA, FLA, and F*A*_1_^′^ modes are marked by red, blue, and gray arrows, respectively. The dashed lines correspond to the peak position of second-order Raman modes, and the *E*′ and *A*_1_^′^ modes are also marked out. (b, c) Comparison between the calculated (Cal, lines) and experimental (Exp, circles) frequencies of twist-angle-dependent Moiré phonons including (b) FTA, FLA, and (c) F*A*_1_^′^ phonons.

Another possible application of our methodology could be the optimization of the large-scale growth for bilayer atomic materials (as shown in Figure S17). In addition, the exfoliated flakes with regular shape could also be the next possible applications of our model (as shown in Figure S18).^56,57^ This thorough workflow acts as a clear guide, detailing the methodology utilized in the study for the precise identification and analysis of twisted bilayers in TMDs grown via CVD. This method can also be adapted to include graphene (as shown in Figure S15) and hBN, as well as their heterostructures.

In conclusion, we have demonstrated a robust and efficient methodology for the identification and analysis of twisted bilayers in TMDs by using deep learning and OpenCV techniques. Our approach, which combines optical micrographs, deep learning models, and OpenCV, provides accurate and comprehensive predictions of the thickness properties and twist angles of bilayer TMDs. The comparison of various deep learning models revealed that the U-Net model exhibits superior performance in terms of global accuracy, mean intersection over union, and processing speed.

Our methodology can be extended to other two-dimensional materials grown by CVD, including both homostructures and heterostructures, highlighting its versatility and broad applicability in the field of 2D materials analysis. Our data sets and codes are made freely available as a service to the community. We hope that, by facilitating and automating the structural analysis of TMDs, this work will contribute to further advancements in the field of TMDs and 2D materials in general and thus also the rapid growing Autonomous lab using AI.^58^

## Methods

A description of the data pipeline, data preparation, deep learning training of the semantic segmentation model, and twist angle identification by OpenCV can be found in the SI.

## Supporting Information Available

Data set preparation, the detailed results of the semantic segmentation, the synthetic data sets, the verification of the twisted bilayer graphene, and the Raman spectra of CVD-grown twisted bilayer MoS_2_. All scripts used in this study are available on GitHub^59^ (PDF)

## References

[ref1] Cao Y.; Fatemi V.; Fang S.; Watanabe K.; Taniguchi T.; Kaxiras E.; Jarillo-Herrero P. Unconventional superconductivity in magic-angle graphene superlattices. Nature 2018, 556, 43–50. 10.1038/nature26160. 29512651

[ref2] Cao Y.; Fatemi V.; Demir A.; Fang S.; Tomarken S. L.; Luo J. Y.; Sanchez-Yamagishi J. D.; Watanabe K.; Taniguchi T.; Kaxiras E.; Ashoori R. C.; Jarillo-Herrero P. Correlated insulator behaviour at half-filling in magic-angle graphene superlattices. Nature 2018, 556, 80–84. 10.1038/nature26154. 29512654

[ref3] Tang Y.; Li L.; Li T.; Xu Y.; Liu S.; Barmak K.; Watanabe K.; Taniguchi T.; MacDonald A. H.; Shan J.; Mak K. F. Simulation of Hubbard model physics in WSe_2_/WS_2_ moiré superlattices. Nature 2020, 579, 353–358. 10.1038/s41586-020-2085-3. 32188950

[ref4] Wu F.; Lovorn T.; Tutuc E.; MacDonald A. H. Hubbard model physics in transition metal dichalcogenide moiré bands. Phys. Rev. Lett. 2018, 121, 026402 10.1103/PhysRevLett.121.026402. 30085734

[ref5] Wang L.; et al. Correlated electronic phases in twisted bilayer transition metal dichalcogenides. Nat. Mater. 2020, 19, 861–866. 10.1038/s41563-020-0708-6. 32572205

[ref6] Devakul T.; Crépel V.; Zhang Y.; Fu L. Magic in twisted transition metal dichalcogenide bilayers. Nat. Commun. 2021, 12, 6730 10.1038/s41467-021-27042-9. 34795273 PMC8602625

[ref7] Lau C. N.; Bockrath M. W.; Mak K. F.; Zhang F. Reproducibility in the fabrication and physics of moiré materials. Nature 2022, 602, 41–50. 10.1038/s41586-021-04173-z. 35110759

[ref8] Quan J.; et al. Phonon renormalization in reconstructed MoS_2_ moiré superlattices. Nat. Mater. 2021, 20, 1100–1105. 10.1038/s41563-021-00960-1. 33753933

[ref9] Marcellina E.; Liu X.; Hu Z.; Fieramosca A.; Huang Y.; Du W.; Liu S.; Zhao J.; Watanabe K.; Taniguchi T.; Xiong Q. Evidence for moiré trions in twisted MoSe_2_ homobilayers. Nano Lett. 2021, 21, 4461–4468. 10.1021/acs.nanolett.1c01207. 33970625

[ref10] Lin K.-Q.; Faria Junior P. E.; Bauer J. M.; Peng B.; Monserrat B.; Gmitra M.; Fabian J.; Bange S.; Lupton J. M. Twist-angle engineering of excitonic quantum interference and optical nonlinearities in stacked 2D semiconductors. Nat. Commun. 2021, 12, 1553 10.1038/s41467-021-21547-z. 33692339 PMC7946969

[ref11] Peimyoo N.; Deilmann T.; Withers F.; Escolar J.; Nutting D.; Taniguchi T.; Watanabe K.; Taghizadeh A.; Craciun M. F.; Thygesen K. S.; Russo S. Electrical tuning of optically active interlayer excitons in bilayer MoS_2_. Nat. Nanotechnol. 2021, 16, 888–893. 10.1038/s41565-021-00916-1. 34083771

[ref12] Sun L.; Wang Z.; Wang Y.; Zhao L.; Li Y.; Chen B.; Huang S.; Zhang S.; Wang W.; Pei D.; Fang H.; Zhong S.; Liu H.; Zhang J.; et al. Hetero-site nucleation for growing twisted bilayer graphene with a wide range of twist angles. Nat. Commun. 2021, 12, 2391 10.1038/s41467-021-22533-1. 33888688 PMC8062483

[ref13] Xie Y.; Wang Z.; Zhan Y.; Zhang P.; Wu R.; Jiang T.; Wu S.; Wang H.; Zhao Y.; Nan T.; Ma X. Controllable growth of monolayer MoS_2_ by chemical vapor deposition via close MoO_2_ precursor for electrical and optical applications. Nanotechnology 2017, 28, 084001 10.1088/1361-6528/aa5439. 27981955

[ref14] Wang Z.; Xie Y.; Wang H.; Wu R.; Nan T.; Zhan Y.; Sun J.; Jiang T.; Zhao Y.; Lei Y.; Yang M.; Wang W.; Zhu Q.; Ma X.; Hao Y. NaCl-assisted one-step growth of MoS_2_–WS_2_ in-plane heterostructures. Nanotechnology 2017, 28, 325602 10.1088/1361-6528/aa6f01. 28718451

[ref15] Paradisanos I.; Shree S.; George A.; Leisgang N.; Robert C.; Watanabe K.; Taniguchi T.; Warburton R. J.; Turchanin A.; Marie X.; Gerber I. C.; Urbaszek B. Controlling interlayer excitons in MoS_2_ layers grown by chemical vapor deposition. Nat. Commun. 2020, 11, 2391 10.1038/s41467-020-16023-z. 32404912 PMC7220905

[ref16] Zhang X.; Nan H.; Xiao S.; Wan X.; Gu X.; Du A.; Ni Z.; Ostrikov K. Transition metal dichalcogenides bilayer single crystals by reverse-flow chemical vapor epitaxy. Nat. Commun. 2019, 10, 598 10.1038/s41467-019-08468-8. 30723204 PMC6363754

[ref17] Wang Z.; Sun J.; Wang H.; Lei Y.; Xie Y.; Wang G.; Zhao Y.; Li X.; Xu H.; Yang X.; Feng L.; Ma X. 2H/1T′ phase WS_2_(1 – *x*)Te_2*x*_ alloys grown by chemical vapor deposition with tunable band structures. Appl. Surf. Sci. 2020, 504, 144371 10.1016/j.apsusc.2019.144371.

[ref18] Dumcenco D.; Ovchinnikov D.; Marinov K.; Lazic P.; Gibertini M.; Marzari N.; Sanchez O. L.; Kung Y.-C.; Krasnozhon D.; Chen M.-W.; Bertolazzi S.; Gillet P.; Morral A. F. i.; Radenovic A.; Kis A. Large-area epitaxial monolayer MoS_2_. ACS Nano 2015, 9, 4611–4620. 10.1021/acsnano.5b01281. 25843548 PMC4415455

[ref19] Yin X.; Ye Z.; Chenet D. A.; Ye Y.; O’Brien K.; Hone J. C.; Zhang X. Edge Nonlinear Optics on a MoS_2_ Atomic Monolayer. Science 2014, 344, 488–490. 10.1126/science.1250564. 24786072

[ref20] Kumar N.; Najmaei S.; Cui Q.; Ceballos F.; Ajayan P. M.; Lou J.; Zhao H. Second harmonic microscopy of monolayer MoS_2_. Phys. Rev. B 2013, 87, 161403 10.1103/PhysRevB.87.161403.

[ref21] Castellanos-Gomez A.; Buscema M.; Molenaar R.; Singh V.; Janssen L.; Van Der Zant H. S.; Steele G. A. Deterministic transfer of two-dimensional materials by all-dry viscoelastic stamping. 2D Mater. 2014, 1, 011002 10.1088/2053-1583/1/1/011002.

[ref22] Wang L.; Meric I.; Huang P.; Gao Q.; Gao Y.; Tran H.; Taniguchi T.; Watanabe K.; Campos L.; Muller D.; Guo J.; Kim P.; Hone J.; Shepard K.; Dean C. One-dimensional electrical contact to a two-dimensional material. Science 2013, 342, 614–617. 10.1126/science.1244358. 24179223

[ref23] Cheng J.; Jiang T.; Ji Q.; Zhang Y.; Li Z.; Shan Y.; Zhang Y.; Gong X.; Liu W.; Wu S. Kinetic nature of grain boundary formation in as-grown MoS_2_ monolayers. Adv. Mater. 2015, 27, 4069–4074. 10.1002/adma.201501354. 26058724

[ref24] Carrascoso F.; Lin D.-Y.; Frisenda R.; Castellanos-Gomez A. Biaxial strain tuning of interlayer excitons in bilayer MoS_2_. J. Phys. Materials 2020, 3, 015003 10.1088/2515-7639/ab4432.

[ref25] Tan P. H.; Han W. P.; Zhao W. J.; Wu Z. H.; Chang K.; Wang H.; Wang Y. F.; Bonini N.; Marzari N.; Pugno N.; Savini G.; Lombardo A.; Ferrari A. C. The shear mode of multilayer graphene. Nat. Mater. 2012, 11, 294–300. 10.1038/nmat3245. 22306771

[ref26] Zhang X.; Han W. P.; Wu J. B.; Milana S.; Lu Y.; Li Q. Q.; Ferrari A. C.; Tan P. H. Raman spectroscopy of shear and layer breathing modes in multilayer MoS_2_. Phys. Rev. B 2013, 87, 115413 10.1103/PhysRevB.87.115413.

[ref27] van der Zande A. M.; Kunstmann J.; Chernikov A.; Chenet D. A.; You Y.; Zhang X.; Huang P. Y.; Berkelbach T. C.; Wang L.; Zhang F.; Hybertsen M. S.; Muller D. A.; Reichman D. R.; Heinz T. F.; Hone J. C. Tailoring the Electronic Structure in Bilayer Molybdenum Disulfide via Interlayer Twist. Nano Lett. 2014, 14, 3869–3875. 10.1021/nl501077m. 24933687

[ref28] Lin M.-L.; Tan Q.-H.; Wu J.-B.; Chen X.-S.; Wang J.-H.; Pan Y.-H.; Zhang X.; Cong X.; Zhang J.; Ji W.; Hu P.-A.; Liu K.-H.; Tan P.-H. Moiré Phonons in Twisted Bilayer MoS_2_. ACS Nano 2018, 12, 8770–8780. 10.1021/acsnano.8b05006. 30086224

[ref29] Wu H.; Lin M.-L.; Zhou Y.; Zhang X.; Tan P.-H. Analyzing Fundamental Properties of Two-Dimensional Materials by Raman Spectroscopy from Microscale to Nanoscale. Anal. Chem. 2023, 95, 10821–10838. 10.1021/acs.analchem.3c00272. 37427912

[ref30] Yoo H.; Engelke R.; Carr S.; Fang S.; Zhang K.; Cazeaux P.; Sung S. H.; Hovden R.; Tsen A. W.; Taniguchi T.; Watanabe K.; Yi G.-C.; Kim M.; Luskin M.; et al. Atomic and electronic reconstruction at the van der Waals interface in twisted bilayer graphene. Nat. Mater. 2019, 18, 448–453. 10.1038/s41563-019-0346-z. 30988451

[ref31] Liu H.; Zheng H.; Yang F.; Jiao L.; Chen J.; Ho W.; Gao C.; Jia J.; Xie M. Line and point defects in MoSe_2_ bilayer studied by scanning tunneling microscopy and spectroscopy. ACS Nano 2015, 9, 6619–6625. 10.1021/acsnano.5b02789. 26051223

[ref32] Li H.; Zhang Q.; Yap C. C. R.; Tay B. K.; Edwin T. H. T.; Olivier A.; Baillargeat D. From bulk to monolayer MoS_2_: evolution of Raman scattering. Adv. Funct. Mater. 2012, 22, 1385–1390. 10.1002/adfm.201102111.

[ref33] Castellanos-Gomez A.; Agraït N.; Rubio-Bollinger G. Optical identification of atomically thin dichalcogenide crystals. Appl. Phys. Lett. 2010, 96, 213116 10.1063/1.3442495.

[ref34] Frisenda R.; Castellanos-Gomez A. Robotic assembly of artificial nanomaterials. Nat. Nanotechnol. 2018, 13, 441–442. 10.1038/s41565-018-0156-5. 29760521

[ref35] Masubuchi S.; Morimoto M.; Morikawa S.; Onodera M.; Asakawa Y.; Watanabe K.; Taniguchi T.; Machida T. Autonomous robotic searching and assembly of two-dimensional crystals to build van der Waals superlattices. Nat. Commun. 2018, 9, 1413 10.1038/s41467-018-03723-w. 29650955 PMC5897399

[ref36] Lin X.; Si Z.; Fu W.; Yang J.; Guo S.; Cao Y.; Zhang J.; Wang X.; Liu P.; Jiang K.; Zhao W. Intelligent identification of two-dimensional nanostructures by machine-learning optical microscopy. Nano Res. 2018, 11, 6316–6324. 10.1007/s12274-018-2155-0.

[ref37] Masubuchi S.; Machida T. Classifying optical microscope images of exfoliated graphene flakes by data-driven machine learning. npj 2D Mater. Appl. 2019, 3, 4 10.1038/s41699-018-0084-0.

[ref38] Han B.; Lin Y.; Yang Y.; Mao N.; Li W.; Wang H.; Yasuda K.; Wang X.; Fatemi V.; Zhou L.; Wang J. I.-J.; Ma Q.; Cao Y.; Rodan-Legrain D.; et al. Deep-learning-enabled fast optical identification and characterization of 2D materials. Adv. Mater. 2020, 32, 2000953 10.1002/adma.202000953. 32519397

[ref39] Masubuchi S.; Watanabe E.; Seo Y.; Okazaki S.; Sasagawa T.; Watanabe K.; Taniguchi T.; Machida T. Deep-learning-based image segmentation integrated with optical microscopy for automatically searching for two-dimensional materials. npj 2D Mater. Appl. 2020, 4, 3 10.1038/s41699-020-0137-z.

[ref40] Sterbentz R. M.; Haley K. L.; Island J. O. Universal image segmentation for optical identification of 2D materials. Sci. Rep. 2021, 11, 5808 10.1038/s41598-021-85159-9. 33707609 PMC7970966

[ref41] Open Source Computer Vision Library. https://opencv.org/ (accessed February 10, 2024).

[ref42] Chen L.-C.; Papandreou G.; Schroff F.; Adam H. Rethinking atrous convolution for semantic image segmentation. ArXiv 2017.

[ref43] Long J.; Shelhamer E.; Darrell T. Fully convolutional networks for semantic segmentation. 2015 IEEE Conference on Computer Vision and Pattern Recognition (CVPR); Boston, MA, USA, 2015; pp 3431–3440. 10.1109/TPAMI.2016.2572683 27244717

[ref44] Howard A.; Sandler M.; Chu G.; Chen L.-C.; Chen B.; Tan M.; Wang W.; Zhu Y.; Pang R.; Vasudevan V.; Le Q. V.; Adam H. Searching for mobilenetv3. 2019 IEEE/CVF International Conference on Computer Vision (ICCV); Seoul, Korea (South), 2019; pp 1314–1324.

[ref45] Ronneberger O.; Fischer P.; Brox T. U-net: Convolutional networks for biomedical image segmentation. Medical Image Computing and Computer-Assisted Intervention–MICCAI 2015:18th International Conference 2015, 18, 234–241. 10.1007/978-3-319-24574-4_28.

[ref46] TorchVision. PyTorch’s Computer Vision Library. https://github.com/pytorch/vision, 2016 (accessed February 10, 2024).

[ref47] He K.; Zhang X.; Ren S.; Sun J. Deep residual learning for image recognition. 2016 IEEE Conference on Computer Vision and Pattern Recognition (CVPR) 2016, 770–778. 10.1109/CVPR.2016.90.

[ref48] Lau C. N.; Bockrath M. W.; Mak K. F.; Zhang F. Reproducibility in the fabrication and physics of moiré materials. Nature 2022, 602, 41–50. 10.1038/s41586-021-04173-z. 35110759

[ref49] Lin K.-Q.; Holler J.; Bauer J. M.; Parzefall P.; Scheuck M.; Peng B.; Korn T.; Bange S.; Lupton J. M.; Schüller C. Large-scale mapping of moiré superlattices by hyperspectral Raman imaging. Adv. Mater. 2021, 33, 2008333 10.1002/adma.202008333. PMC11469034 34242447

[ref50] Lu A.-Y.; Martins L. G. P.; Shen P.-C.; Chen Z.; Park J.-H.; Xue M.; Han J.; Mao N.; Chiu M.-H.; Palacios T.; Tung V.; Kong J. Unraveling the Correlation between Raman and Photoluminescence in Monolayer MoS_2_ through Machine-Learning Models. Adv. Mater. 2022, 34, 2202911 10.1002/adma.202202911. 35790036

[ref51] Yang R.; Yousuf S. E. H.; Lee J.; Zhang P.; Liu Z.; Feng P. X.-L. Raman spectroscopic probe for nonlinear MoS_2_ nanoelectromechanical resonators. Nano Lett. 2022, 22, 5780–5787. 10.1021/acs.nanolett.2c01250. 35792575

[ref52] Xie Y.; Lee J.; Wang Y.; Feng P. X.-L. Straining and Tuning Atomic Layer Nanoelectromechanical Resonators via Comb-Drive MEMS Actuators. Advanced Materials Technologies 2021, 6, 2000794 10.1002/admt.202000794.

[ref53] Hu W.; Wang Y.; He K.; He X.; Bai Y.; Liu C.; Zhou N.; Wang H.; Li P.; Ma X.; Xie Y. Straining of atomically thin WSe_2_ crystals: Suppressing slippage by thermal annealing. J. Appl. Phys. 2022, 132, 085104 10.1063/5.0096190.

[ref54] Sun Y.; Yin S.; Peng R.; Liang J.; Cong X.; Li Y.; Li C.; Wang B.; Lin M.-L.; Tan P.-H.; Wan C.; Liu K. Abnormal out-of-plane vibrational Raman mode in electrochemically intercalated multilayer MoS_2_. Nano Lett. 2023, 23, 5342–5349. 10.1021/acs.nanolett.3c01543. 37219946

[ref55] Zhang X.; Qiao X.-F.; Shi W.; Wu J.-B.; Jiang D.-S.; Tan P.-H. Phonon and Raman scattering of two-dimensional transition metal dichalcogenides from monolayer, multilayer to bulk material. Chem. Soc. Rev. 2015, 44, 2757–2785. 10.1039/C4CS00282B. 25679474

[ref56] Palai S. K.; Dyksik M.; Sokolowski N.; Ciorga M.; Sánchez Viso E.; Xie Y.; Schubert A.; Taniguchi T.; Watanabe K.; Maude D. K.; Surrente A.; Baranowski M.; Castellanos-Gomez A.; Munuera C.; Plochocka P. Approaching the Intrinsic Properties of Moiré Structures Using Atomic Force Microscopy Ironing. Nano Lett. 2023, 23, 4749–4755. 10.1021/acs.nanolett.2c04765. 37276177 PMC10273312

[ref57] Zhang N.; Surrente A.; Baranowski M.; Maude D. K.; Gant P.; Castellanos-Gomez A.; Plochocka P. Moiré intralayer excitons in a MoSe_2_/MoS_2_ heterostructure. Nano Lett. 2018, 18, 7651–7657. 10.1021/acs.nanolett.8b03266. 30403876

[ref58] Ren Z.; Ren Z.; Zhang Z.; Buonassisi T.; Li J. Autonomous experiments using active learning and AI. Nat. Rev. Mater. 2023, 8, 563–564. 10.1038/s41578-023-00588-4.

[ref59] Xie Y. Twist2DNet. https://github.com/YongXie-ICMM/Twist2DNet.git, 2024 (accessed February 10, 2024).

