# Supplementary material for: Identification and Structural Characterization of Twisted Atomically Thin Bilayer Materials by Deep Learning
Source: Nano Lett. 2024 Feb 26;24(9):2789–97. doi: 10.1021/acs.nanolett.3c04815 (PMC10921996; doi:10.1021/acs.nanolett.3c04815)
Supplement: Supplementary file 1 — nl3c04815_si_001.pdf [file nl3c04815_si_001.pdf]

Supplementary Information:Identification and Structural Characterization of Twisted Atomically Thin Bilayer Materials by Deep Learning

Haitao Yang,[1,*] Ruiqi Hu,[2,*] Heng Wu,[3,*] Xiaolong He,[1,*] Yan Zhou,[3,4,†] Yizhe Xue,[1] Kexin He,[1] Wenshuai Hu,[1] Haosen Chen,[1] Mingming Gong,[5] Xin Zhang,[3] Ping-Heng Tan,[3,‡] Eduardo R. Hernández,[6,§] and Yong Xie[1,6,¶]

[1]*Key Laboratory of Wide Band-Gap Semiconductor Technology & Shaanxi Key Laboratory of High-Orbits-Electron Materials and Protection Technology for Aerospace, School of Advanced Materials and Nanotechnology, Xidian University, Xi'an 710071, China*

[2]*Department of Materials Science and Engineering, University of Delaware, Newark, DE 19716, USA*

[3]*State Key Laboratory of Superlattices and Microstructures, Institute of Semiconductors, Chinese Academy of Sciences, Beijing 100083, China*

[4]*Phonon Engineering Research Center of Jiangsu Province, School of Physics and Technology, Nanjing Normal University, Nanjing 210023, China.*

[5]*School of Materials Science and Engineering, Northwestern Polytechnical University, Xi'an 710072, China*

[6]*Instituto de Ciencia de Materiales de Madrid (ICMM-CSIC), 28049 Madrid, Spain*

Supplementary Information

Workflow

1. Experimental Section/Methods

1.1. Datasets preparation (CVD and data preprocessing)

1.2. First deep learning training

1.3. OpenCV calculation for twisted bilayer materials

1.4. Artificial/Synthetic datasets generation and the second deep learning training

1.5. Second-harmonic generation (SHG) measurements

1.6. Raman spectroscopy

2. Discussion

2.1. First deep learning model performance

2.2. Twist angle calculation by OpenCV

2.3. Prediction of the bilayer Graphene twist angles by deep learning

2.4. Raman spectroscopy results

Supplementary Figures

Reference

Workflow

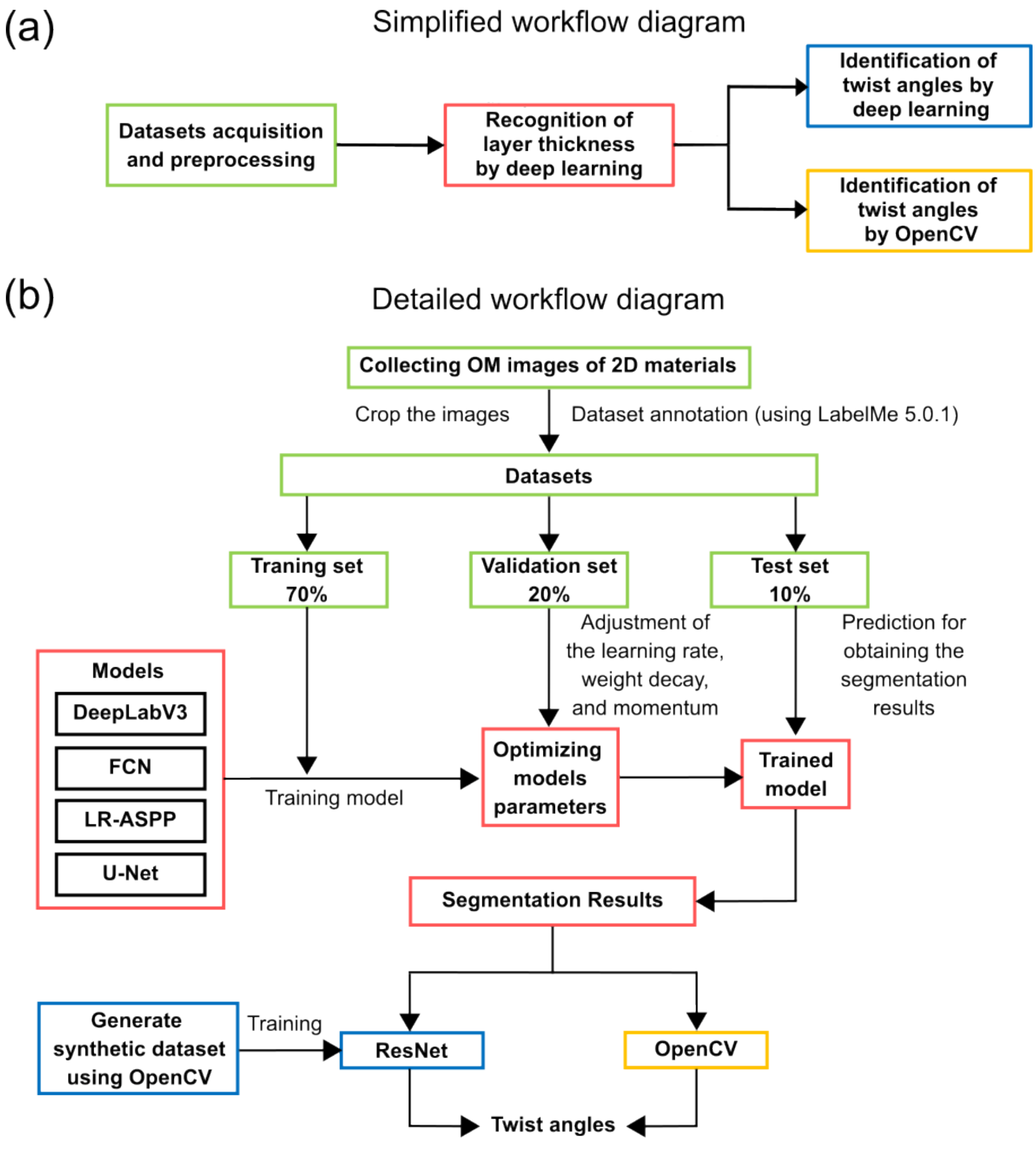

FIG. S1. **Workflow diagram of the process to identify the twist bilayer of TMDs** (a) Simplified version of the workflow diagram with details in (b). The green, red and blue color illustrate three different steps of the identification process. Procedures for datasets acquisition and processing, as well as the recognition of layer thickness using deep learning, are all elaborated in Section 1. More information relates to the method to calculate twist angles by OpenCV is shown in Section 1.

1. Experimental Section/Methods

All scripts used in this study are available on GitHub [1]. Currently, the scripts code is provided as a supplementary file for reviewers and others to examine. Upon acceptance of the paper, it will be made open-source on the corresponding GitHub repository. Each subfolder in the supplementary file specifically correlates with the respective sections of the current supplementary section.

1.1 Datasets preparation (CVD and data preprocessing)

1.1.1 CVD growth

TMDs were grown using a modified CVD process, as shown in our previous reports [2–4]. First, the metal source, such as MoS_2 powder, was loaded into the boat with the SiO_2 / Si substrate facing the source materials. The source boat was placed at the center area of the single-zone furnace. Then, the sulfur powder was loaded onto the edge of the furnace with a movable inner tube and heated above 100 °C for more than 15 min to eliminate the water inside before growth. The furnace was then heated up to 750–800 °C with a heating rate of 25–30 °C/min and kept at the growth temperature for 3–5 min. After growth, the furnace was cooled down undisturbed to under 300 °C and then was opened for fast cooling to below 100 °C and the substrate was removed. An optical micrograph was captured using a Leica microscope (DM2700 M). After growth, images (in tiff format) with a resolution of 2592×1944 pixels were captured with the microscope.

1.1.2 Crop images

To augment our datasets, we utilized Python to crop the 69 original micrograph images, each of resolution 2592×1944, yielding a total of 1035 images sized 512×512, along with their respective color maps. Each image was saved in the .jpg format, adhering to the naming convention MoS_2_i. Fig. S3(a) showcases an optical micrograph of the as-grown CVD MoS_2, while Fig. S3(b) displays a cropped section of the same micrograph for clearer analysis.

1.1.3 Annotation using LabelMe 5.0.1

To annotate the optical micrographs, we opted for the **LabelMe 5.0.1** software due to its robustness and user-friendly interface for such tasks. The process begins with the software's main interface, as depicted in Fig. S4(a). The annotation of original images is then completed by following the sequential steps illustrated in Fig. S4(b), resulting in the generation of a JSON file.

Specifically, the steps shown in Fig. S4(b) start with utilizing the *Open Dir* function, allowing users to navigate to and select the directory containing the images to be annotated. Once the directory is selected, the images are loaded into the main workspace in sequential order for annotation.

After loading the images, the *Create Polygons* feature is used to manually trace the boundaries of the CVD samples. This polygonal tracing is highly precise, capturing subtle differences in sample thickness, particularly when focusing on variations in thickness. Properly labeling these delineated regions becomes crucial after completing the tracing of contours. Each outlined region is labeled based on the perceived thickness, typically categorized into three distinct classes: monolayer (**1L**), bilayer (**2L**), or thick layer (**TL**).

Upon completion of annotation and labeling, the annotations, including polygonal boundaries and associated labels, are saved in JSON format using LabelMe's local saving mechanism. The results of the annotation process can be observed in Fig. S5. This JSON format ensures easy retrieval of annotations and compatibility with many deep learning frameworks for subsequent processing and analysis.

1.1.4 Conversion of json annotations to datasets

After annotation, the .json files are converted to the appropriate image format. This conversion is facilitated by tools from LabelMe, specifically by `labelme_json_to_dataset.exe` according to `json_to_dataset.bat`. Each .json file results in four distinct files:

- `image.jpg` - the original image,
- `label.png` - showcasing thickness variations through different colors,
- `label_names.txt` - listing all category names from the LabelMe annotation. Typically, the first line is "_background_", denoting unannotated areas.

- `label_viz.png` - a visual representation of category distribution and color associations.

An exemplary set of datasets, as obtained after converting the json files using the "labelme_json_to_dataset.exe" program in LabelMe, is depicted in Fig. S6. For ensuing steps, the `label.png` files are stored individually, using naming conventions like MoS_2_*i.png*.

1.1.5 Datasets augmentation and storage

With the datasets ready, augmentation techniques are applied to enhance its diversity, reducing potential overfitting in the training process. For structured storage, the VOC2007 [5]format was employed, a benchmark in object detection and image classification contexts. Our annotated datasets are housed within the "VOCdevkit" directory, aligning with the VOC2007 structure. This directory encompasses three chief folders: "ImageSets", "JPEGImages", and "SegmentationClass". "JPEGImages" contains the annotated TMDs micrographs, which after semantic segmentation categorization, are transferred to the "SegmentationClass" folder in PNG format. "ImageSets" contains textual representations for datasets demarcation, with files such as "train.txt", "val.txt", "trainval.txt", and "test.txt" denoting training, validation, combined training-validation, and test datasets respectively. Adhering to a 7:2:1 ratio, the datasets are partitioned using the "Assign_Datasets.py" script.

1.2 First deep learning training

1.2.1 Loss function

The classification of layer thicknesses on a pixel level is a multi-class classification problem (here, four different categories, background, 1L, 2L and TL, are used). Hence, the cross-entropy loss function is used as the standard practice in classification problems. The logarithmic Softmax (LogSoftmax) function is employed to normalize the probability of the predicted category for each pixel in a sample image, mapping it to a range between 0 and 1 as follows:

$$y_j^{(i)} = \text{softmax}(x_j^{(i)}) = \frac{e^{x_j^{(i)}}}{\sum_{k=1}^{C} e^{x_k^{(i)}}}, \tag{1}$$

where $x^{(i)}$ is the input of the predicted category probability for the pixel (here the probability of layer thickness observed at pixel i: background, 1L, 2L, TL). $y_j^{(i)}$ is the resulting probability that pixel i belongs to the j-th class, and C represents the number of classes in

the classification problem (in our case $C = 4$). The cross-entropy loss function is defined as follows:

$$l(\hat{y}, y) = -\frac{1}{N}\sum_{i=1}^{N}\sum_{j=1}^{K}\hat{y}_j^{(i)}\log y_j^{(i)}, \tag{2}$$

where $\hat{y}_j^{(i)}$ represents the true value of the j-th label for pixel i. Assuming that there are C categories in total, the true label of each sample is a C-dimensional vector, where only the j-th element has a value of 1 and the remaining elements are 0. Plugging Eq. (1) into equation Eq. (2) results in the following expression for the loss function:

$$l(\hat{y}, x) = -\frac{1}{N}\sum_{i=1}^{N}\sum_{j=1}^{C}\hat{y}_j^{(i)}\log\left(\frac{e^{x_j^{(i)}}}{\sum_{k=1}^{C} e^{x_k^{(i)}}}\right). \tag{3}$$

Subsequently, the Stochastic Gradient Descent (SGD) algorithm is employed to minimize the cross-entropy loss Eq. (3) by iteratively updating the network's weights so as to reduce the loss function. During training of the AI models tested in this work, standard data augmentation methods were employed to minimize the risk of over-fitting; specifically these were a random re-size of the images, horizontal and vertical flips, and image cropping. More details are presented in the Supplementary Information file.

1.2.2 Training Configuration and Model Evaluation

Following the preparation of the datasets and establishment of the semantic segmentation neural network, the next logical step was setting up the training parameters. For this purpose, Python's "argparse" module was employed, facilitating the efficient integration of parameters into our program.

During the training phase, if results appear to be less than ideal, fine-tuning parameters such as the learning rate, weight decay, and momentum is pivotal. In our framework, default values have been earmarked for these deep learning parameters. The "lr" parameter starts with a default value of 0.015. Both "momentum" and "weight_decay" parameters, integral to the stochastic gradient descent (SGD) method, are initialized to 0.9 and 0.0001 respectively [6]. We've chosen the cross-entropy loss function for our DL model training, a choice traditionally reserved for classification challenges. Throughout the training, a consistent batch size of 4 is used.

For a thorough evaluation, we have utilized four foundational algorithms as depicted in Fig. S7. These algorithms include DeepLabV3 [7], FCN [8], LR-ASPP [9], and U-Net [10].

The specific structures and functionalities of these algorithms can be gleaned from the mentioned figure.

Since all models used here are taken from the literature, it is possible to start the training with the pre-trained weights of each model. However, we have observed that for our particular application this is not necessarily the best course of action.

1.3 OpenCV calculation for twisted bilayer materials

1.3.1 OpenCV calculate twist angle workflow

As illustrated in Fig. S8, we employ OpenCV to identify the twist angles in bilayer MoS_2. The figure delineates a streamlined workflow for the twist angle identification process (a), accompanied by the detailed steps involved in the process from (b) to (h). Initially, the MoS_2 obtained through Chemical Vapor Deposition (CVD) undergoes semantic segmentation (c). Subsequently, MoS_2 flakes of varying thicknesses are categorized (d). The contours of single-layer and bilayer MoS_2 are extracted (e). A mask operation is then executed on the semantically segmented images to derive the spatial information of 2L MoS_2 within the contour of 1L MoS_2 (f). Thereafter, the rotation of the first and second layers of MoS_2 in relation to the center of MoS_2 is computed (g). Finally, the twist angle of the bilayer TMDs is determined (h). This approach offers an efficient and accurate means to analyze the structural attributes of bilayer TMDs.

1.3.2 Extraction of the spatial distribution of each category based on RGB information

In our analysis, each category within the test images (notably "test.png") is meticulously discerned based on its RGB information. We employ the "palette.json" file to define the lower and upper bounds of the RGB channels, which in turn designates the precise color range for every distinct category. This process facilitates a refined categorization by extracting the RGB range, particularly by utilizing the "InRange" function in OpenCV. Once this is achieved, the spatial distribution of each category can be accurately segregated.

1.3.3 Filtering of triangular shapes based on edge and contour information

The outermost contours of each TMD flake can be extracted (using the ”findContours” function in OpenCV), and then the area corresponding to each contour is determined (using the ”contourArea” function in OpenCV). After polygon fitting on the contour (using the ”approxPolyDP” function in OpenCV), we obtained the number of corner points. Since TMDs typically have a triangular morphology, the number of corner points is limited to 3 to preliminarily screen out contour areas with a triangular shape. Then utilized to fit the smallest circumscribed triangle(using the ”minEnclosingTriangle” function in OpenCV) for these contour areas, which yields vertex coordinates, area, and other information. The vertex information is used to derive the inner angle of the smallest circumscribed triangle, which helps to further screen the target object based on its inner angle.

1.3.4 Image processing operations to obtain the twist angle of bilayer TMDs

In order to determine the twist angle of transition metal dichalcogenides (TMDs), the outermost contour of the single-layer triangular shape detected and filled on a temporary image (using the ”drawContours” function in OpenCV). Then extract the bilayer area(using the ”inRange” function in OpenCV), and locate the target triangular shape on the bilayer(using a the ”findContours” function in OpenCV). This process allows for the retrieval of the position information of the entire contour on the micrograph, which can be used to identify the RGB value of the pixel in the temporary image.

By assessing whether the bilayer triangular shape TMDs are grown on a single-layer triangular shape flake, the twist angle can be determined. Using trigonometric functions, the leftmost two points of each fitted triangle are obtained to calculate its angle relative to the Cartesian coordinate system with the center of the image as the origin, which can be used as the distance between each triangular target and the entire image rotation angle. Finally, the absolute value of the subtraction between the rotation angles of the bilayer and single-layer triangular flakes is taken into account as the twist angle value.

1.4 Artificial/Synthetic datasets generation and the second deep learning training

To facilitate the training of our CNN model, we leveraged the capabilities of OpenCV to generate an extensive artificial datasets. This process resulted in the creation of 10144 synthetic images, designed to simulate real-world conditions and to ensure robust model training. The distribution of angles within this datasets is a pivotal aspect, having profound implications for the training dynamics and the subsequent model performance.

Fig. S9 offers a visual representation of the angle distribution within our artificial datasets. This distribution provides insights into the diversity and scope of angles in the images we generated, showcasing the comprehensive nature of our training data. By crafting such a diverse datasets, we are gearing up our model to excel across a spectrum of real-world applications and scenarios.

1.5 Second-harmonic generation (SHG) measurements

The Second Harmonic Generation (SHG) experiments were measured by a Horiba Jobin-Yvon HR800 Raman system equipped with a liquid-nitrogen-cooled charge couple detector (CCD) integrated spectrometer and a $\times 50$ objective lens (numerical aperture = 0.55) mounted microscope, along with a Ti: Sapphire ultrafast laser (Coherent Chameleon Ultra) pumping at 800 nm and operating at an 80 MHz repetition rate under a pulse duration of 140 ± 20 fs.To avoid sample damage by laser heating, the average laser power was kept below 150 μW. A polarizer and a corresponding half-wave plate were placed in the incident path, while an analyzer lying parallel to the polarization direction of the pump beam was placed before the spectrometer to collect the polarization components of the SHG radiation.

Our approach began with conducting SHG measurements on individual monolayers of MoS_2. This initial step was crucial for understanding the intrinsic nonlinear optical characteristics of single-layer MoS_2 by observing the SHG signal generated upon laser excitation. Following this, we focused on bilayer MoS_2 regions where two monolayers overlap. By analyzing the SHG data from each monolayer, we were able to calculate the twist angle in the bilayer configuration by assessing variations in the SHG intensity and pattern.

1.6 Raman spectroscopy

Raman spectra are measured at room temperature using a Horiba Jobin-Yvon HR800 micro-Raman system equipped with a liquid-nitrogen-cooled charge couple detector (CCD), a 1800 lines/mm grating, and a $\times 100$ objective lens (numerical aperture = 0.90). The excitation energy is 2.54 eV from an Ar^{+} laser, which enables each CCD pixel to cover 0.62 cm^{-1}. A typical laser power of 0.3 mW is used to avoid sample heating.

2. Discussion

2.1 First deep learning model performance

Based on Table.S1, the *U-Net* approach surpasses the *DeepLabV3* and *FCN* models in terms of global accuracy by 0.2 % and *LR-ASPP* by 0.9 %. Furthermore, the *U-Net* model demonstrates superior performance in mean intersection over Union. Additionally, even when running solely on CPU, the *U-Net* achieves higher frames-per-second (FPS) processing rates of 3.56, which is higher than that achieved by the *DeepLabV3* (1.16) and *FCN* (1.32) models. The GPU-assisted *U-Net* model exhibits even better performance, being 175 % and 149 % more efficient than *DeepLabV3* and *FCN* models, respectively. Overall, *U-Net* displays a better average accuracy and performs best in background and monolayer detection, followed by bilayer and thick-layer classes.

Upon evaluating the performance of the four models, as evidenced by their respective confusion matrices in Fig. S10, we found that U-Net performs better than the other three models in terms of classification accuracy for BG, 1L, and 2L. Specifically, while (a) *DeepLabV3*, (b) *FCN*, and (c) *LR-ASPP* show certain levels of performance, it is (d) *U-Net* that stands out for BG, 1L, and 2L classifications. Although U-Net slightly lags behind DeepLabV3 in predicting the TL category, considering our subsequent research focus on corners with a special emphasis on 1L and 2L predictions, U-Net becomes our first choice. Its high accuracy ensures more reliable prediction on our subsequent studies.

Semantic segmentation predictions are obtained by using the "predict.py" script. This script categorizes the input image at the pixel level, with each designated category being color-mapped according to the RGB values specified in the "palette.json" file. The processed

result is then saved as "test_result.png". The configuration parameters within the script can be adjusted to tailor the output, yielding segmentation results analogous to those depicted in Fig. S11.

2.2 Twist angle calculation by OpenCV

Leveraging the OpenCV library, we developed a methodology to precisely discern the twist angles in bilayer MoS_2. This process, as elucidated in Fig. S8, commences with the execution of the "get_twist_angel.py" script. The "argparse" module plays a pivotal role, channeling the parameters into our Python script for efficient angle computation.For a more illustrative understanding, Fig. S12 delves deeper into the twist-angle analysis for bilayer MoS_2 from our experimental endeavors.

2.3 Prediction of the bilayer Graphene twist angles by deep learning

In addition to our endeavor with TMDs, we have extended our approach to the realm of hexagonal Graphene structures. Utilizing the same methodology of generating artificial datasets by OpenCV, we trained the ResNet model to predict twist angles in Graphene [11]. As demonstrated in Fig. S14 and Fig. S15, our model exhibits competence in predicting the twist angles of these Graphene structures, reinforcing the versatility and reliability of our method. This underscores the robustness of our approach, indicating its potential for reliable predictions across various two-dimensional materials.

2.4 Raman spectroscopy results

We first concentrate on the results presented in Fig. S16 and Fig.5, which showcase a comparative analysis between angles determined through a two-step deep learning approach and those obtained via manual annotation. This comparison is integral to our study as it evaluates the effectiveness and precision of advanced machine learning techniques in contrast to traditional manual methods in determining structural angles.

Supplementary Figures

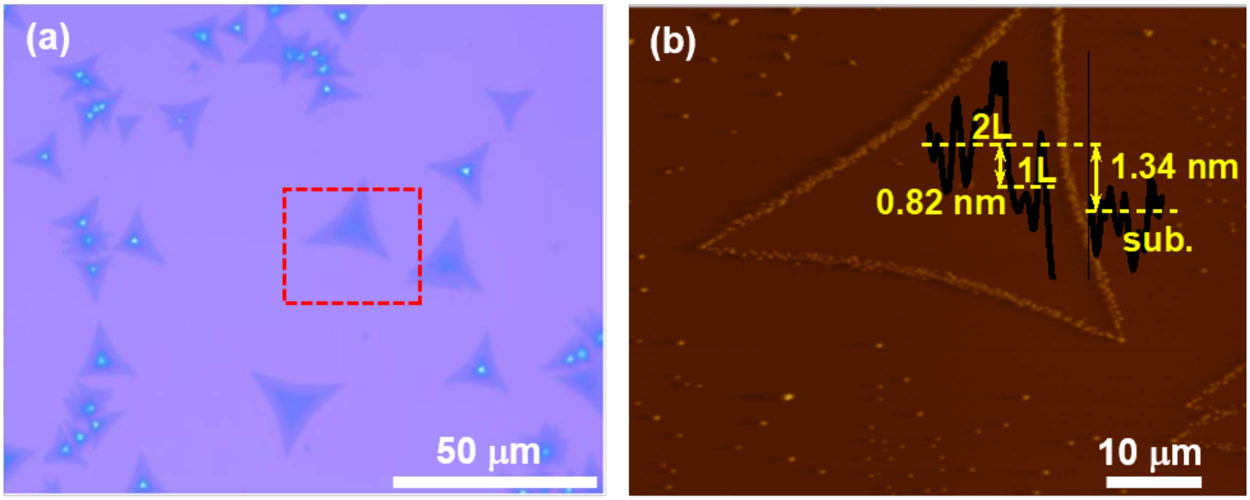

FIG. S2. **Correlation of thickness measurements between optical microscopy and AFM.** Displayed are (a) a typical optical micrograph and (b) AFM topography of MoS_2, featuring areas of single-layer and bilayer thickness. These images clearly delineate the atomically thin step increments, with the single layer exhibiting a thickness of 0.82 nm and the bilayer demonstrating a thickness of 1.34 nm.

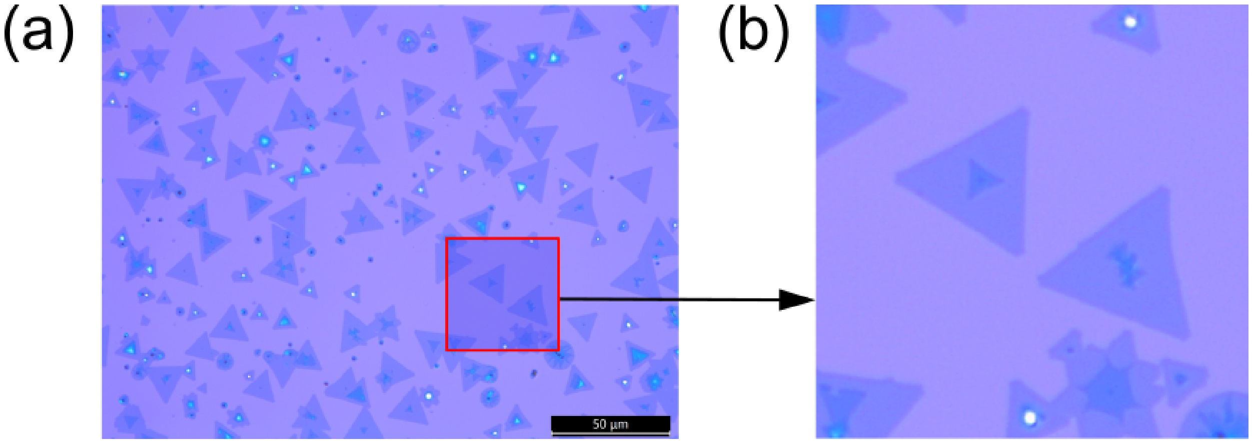

FIG. S3. **Optical mircrograph of as grown samples.** (a) The optical micrograph with a resolution of 2592×1944 and the (b) cropped image with a resolution of 512×512 of the as-grown CVD MoS_2 .

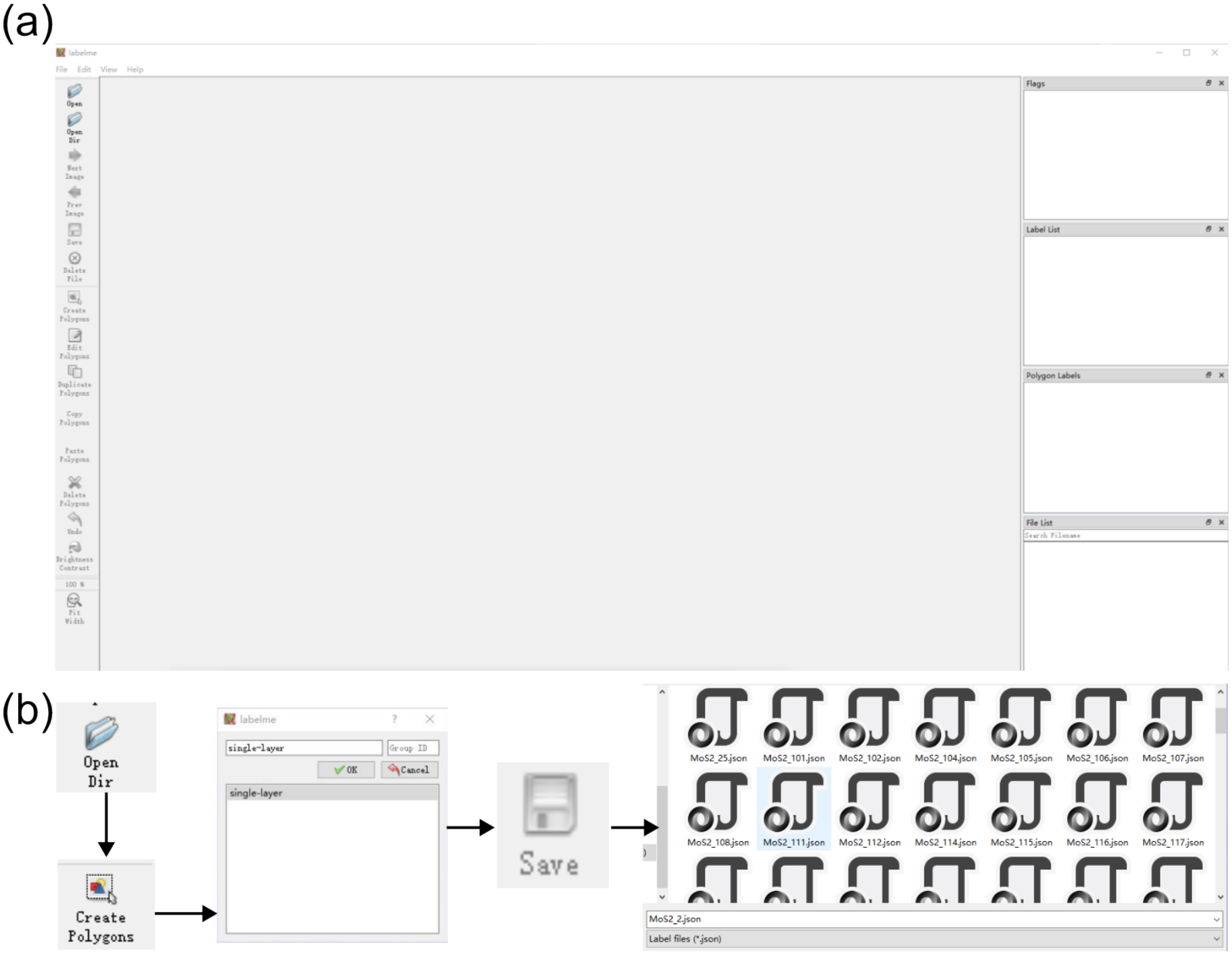

FIG. S4. **Detailed process of labelling.** (a) Main interface of LabelMe. (b) Instruction of the datasets' creation using LabelMe.

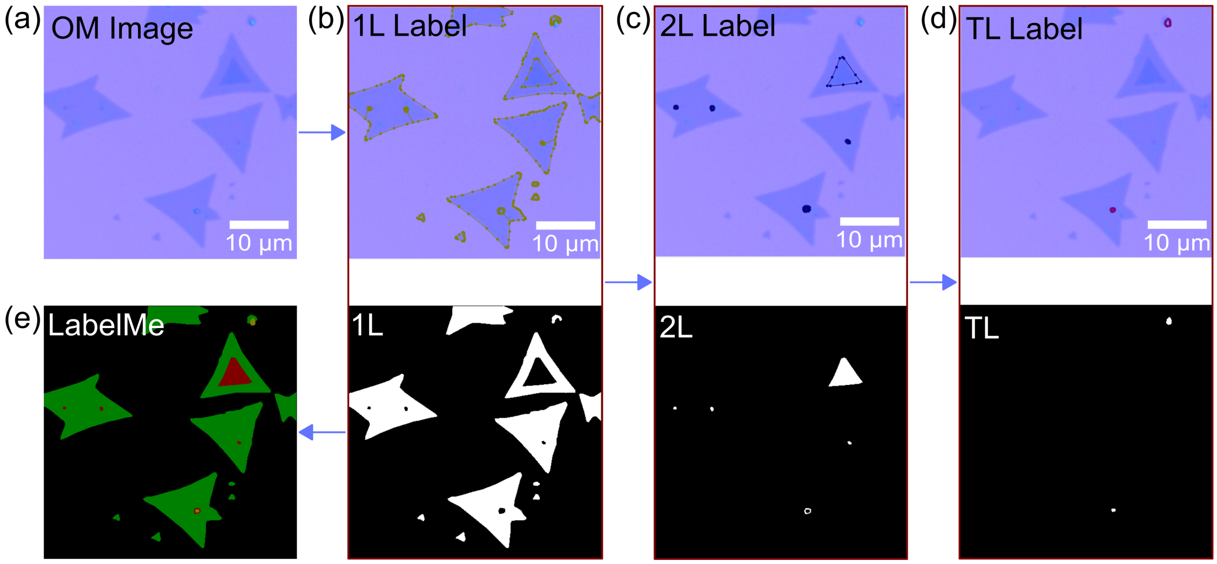

FIG. S5. **Detailed overview of the labeling process as described in Supplementary section 1.1.3.**

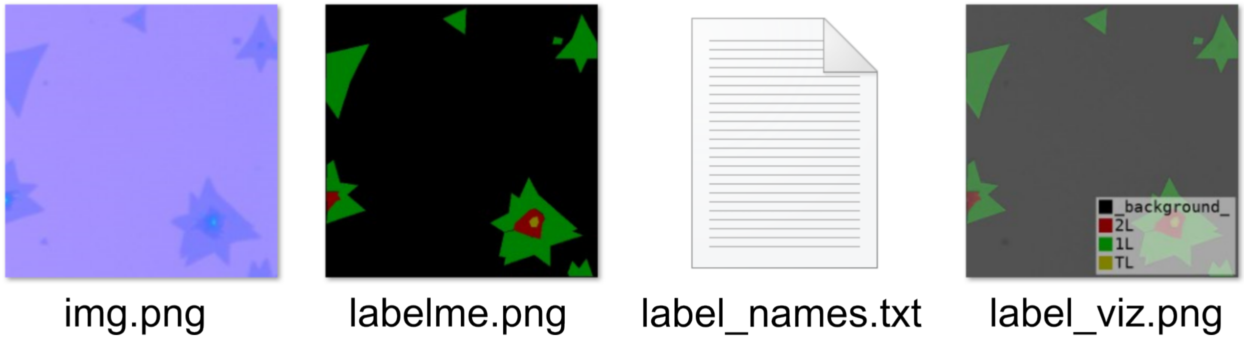

FIG. S6. **An example of the datasets obtained after converting the json files using the "labelme_json_to_dataset.exe" program in LabelMe.**

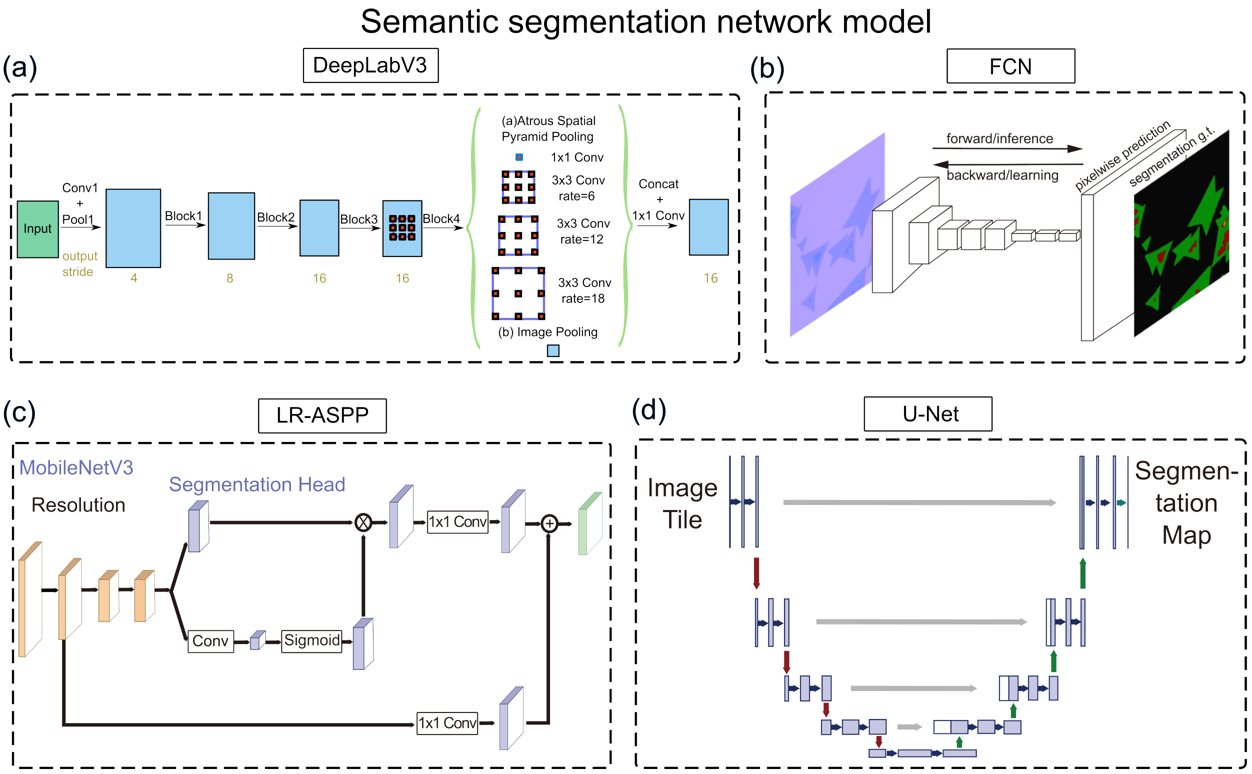

FIG. S7. **Four CNN models used in our identification process.** (a) DeepLabV3, (b) FCN, (c) LR-ASPP and (d) U-Net.

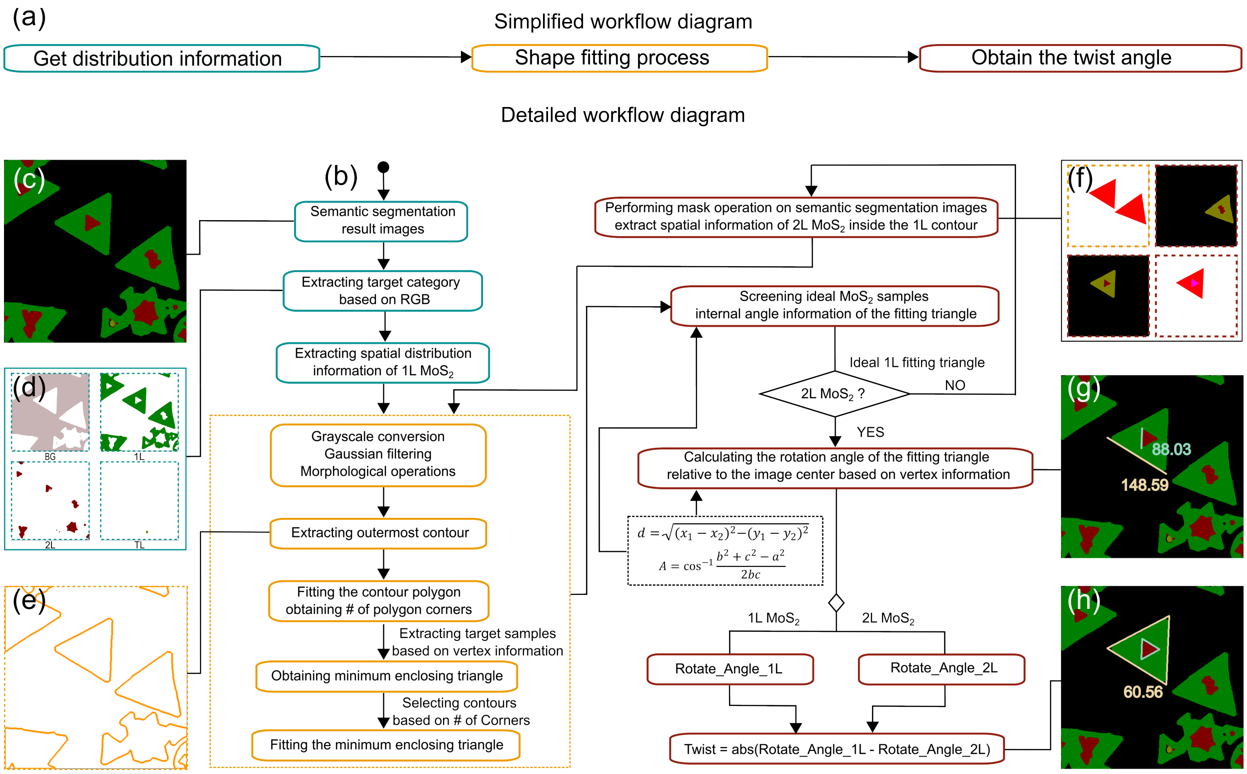

FIG. S8. **Identification of twist angles in bilayer MoS_2 using OpenCV.** (a) A simplified workflow for the twist angle identification process. (b)-(h) Detailed steps of the twist angle identification process. (c) Semantically segmented CVD MoS_2. (d) MoS_2 flakes of different thicknesses are categorized. (e) Contours of single-layer and bilayer MoS_2 are extracted. (f) Mask operation is performed on the semantic images to extract the spatial information of 2L MoS_2 within the 1L MoS_2 contour. (g) The rotation of the first and second layers MoS_2 with respect to the center of MoS_2 is calculated. (h) The twist angle of the bilayer TMDs is determined.

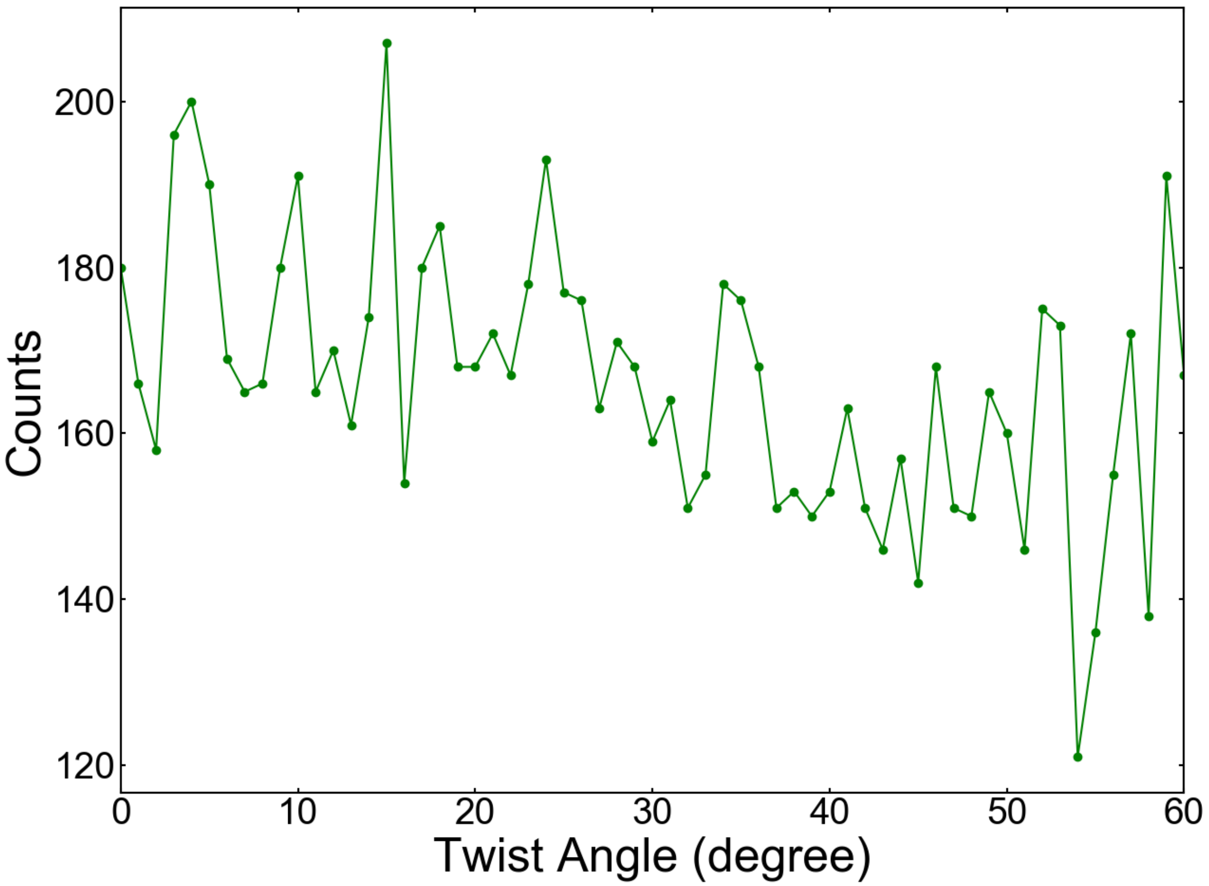

FIG. S9. **Angle distribution of the artificial 10144 dataset files used in the second CNN model.**

TABLE S1. **Classification performance of each semantic segmentation model on the test datasets.** The global accuracy (by pixel), Mean Intersection over Union (by pixel), training time and frames per second (FPS) under two experimental environments, CPU: Intel(R) Core(TM) i7-12700KF CPU @ 3.60GHz, 32.0GB RAM; GPU: NVIDIA GeForce RTX 3080 Ti, 12G GDDR6X are shown. Besides, average accuracy and Intersection over Union (IoU) for the four categories in four models are compared.

Model name		*DeepLabV3*	*FCN*	*LR-ASPP*	*U-Net*
Global accuracy (by pixel)		98.2	98.2	97.5	98.4
Mean Intersection over Union (by pixel)		81.9	81.4	76.6	83.7
Training time		29m24s	25m18s	17m18s	34m18s
Frames per second (FPS)	CPU	1.16	1.32	9.80	3.56
	GPU	45.66	50.35	201.25	125.63
Average accuracy (by pixel)	Background (BG)	99.1	99.2	98.9	99.2
	Monolayer (1L)	93.6	93.3	91.9	93.7
	Bilayer (2L)	84.8	86.4	69.2	89.1
	Thick Layer (TL)	83.2	77.5	71.0	80.8
Intersection over Union (by pixel)	Background (BG)	98.5	98.5	97.9	98.6
	Monolayer (1L)	87.1	87.1	82.4	88.1
	Bilayer (2L)	71.2	70.9	60.1	74.5
	Thick Layer (TL)	70.7	69.2	65.9	73.7

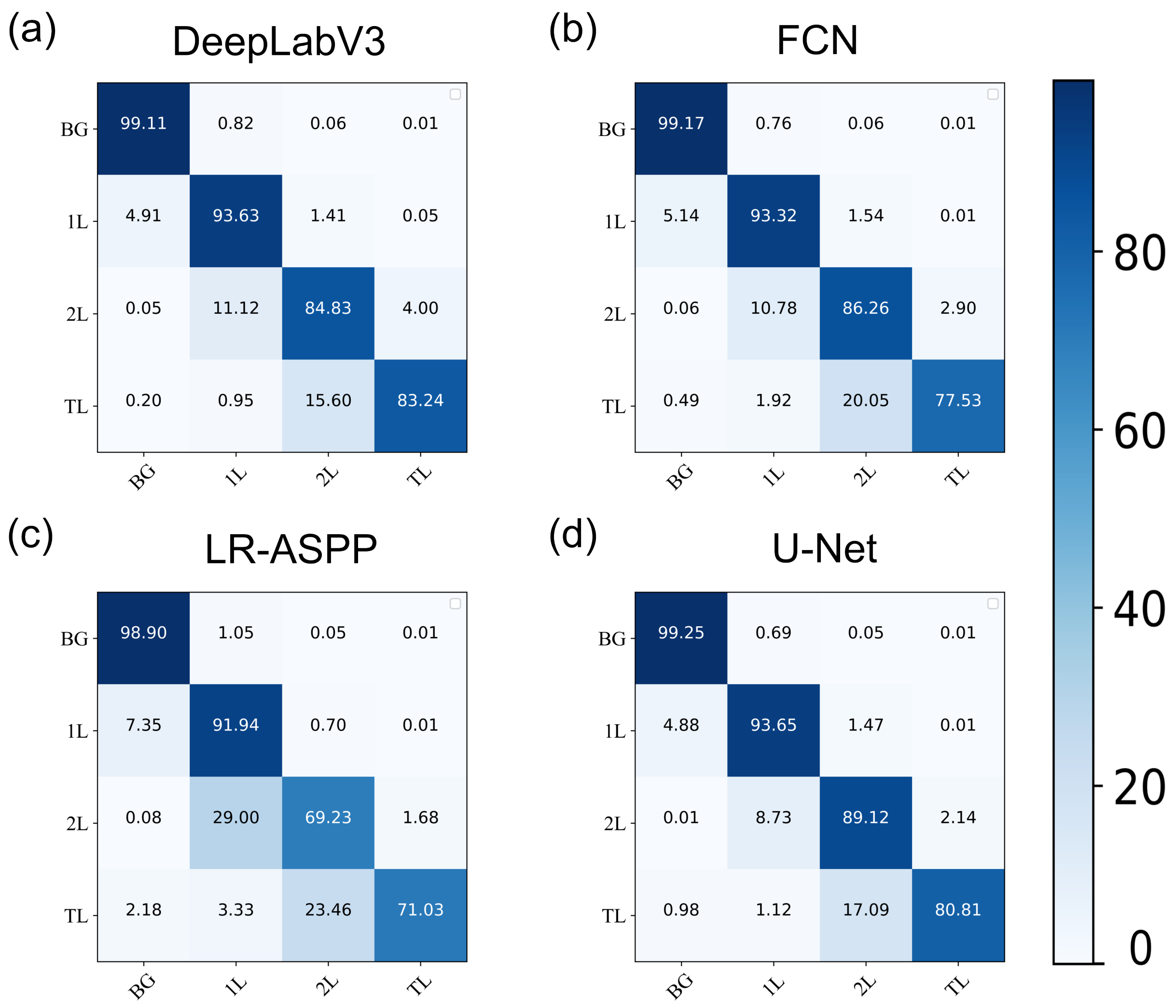

FIG. S10. **Confusion matrices of each class after the semantic segmentation network.** (a) *DeepLabV3*, (b) *FCN*, (c) *LR-ASPP*, (d) *U-Net*.

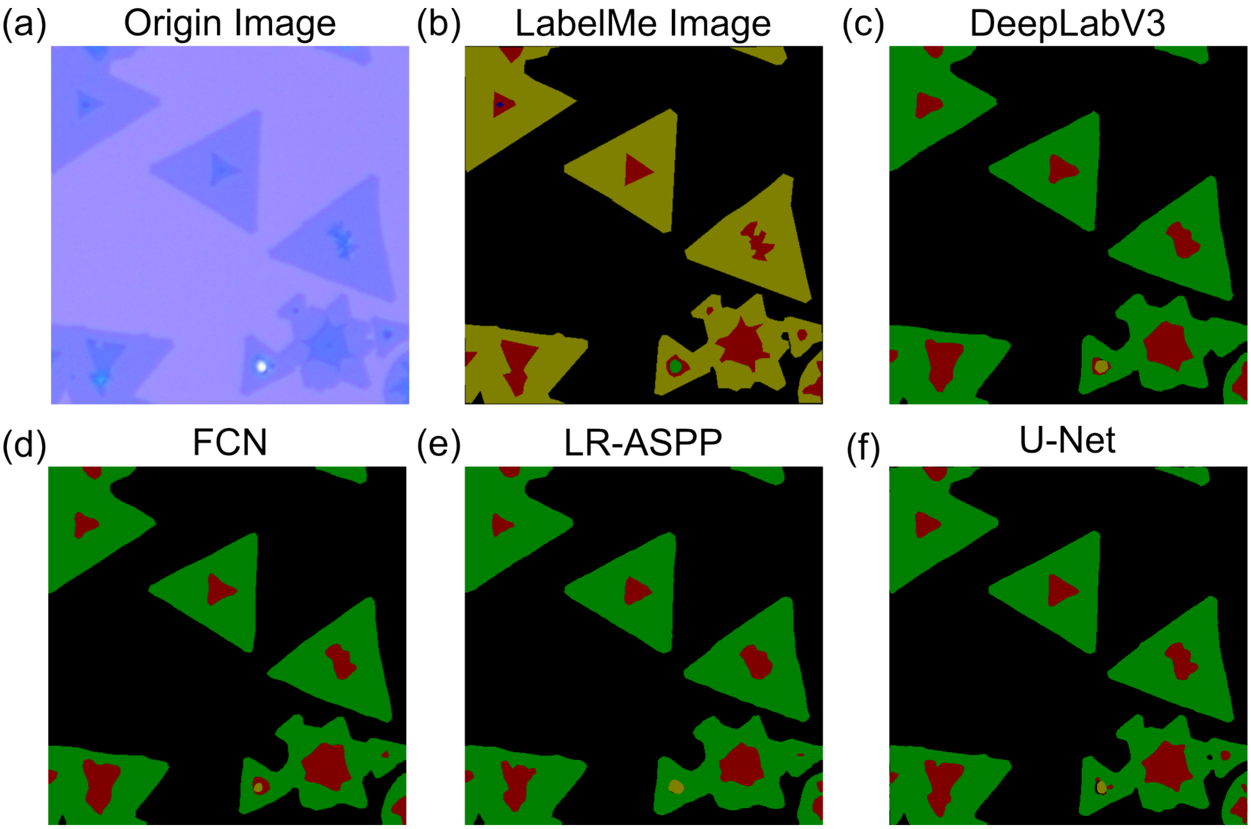

FIG. S11. **Predicted results from different semantic segmentation models.** (a) Origin image. (b) Ground truth image annotated using LabelMe. Predicted images from (c) DeepLabV3, (d) FCN, (e) LR-ASPP and (f) U-Net.

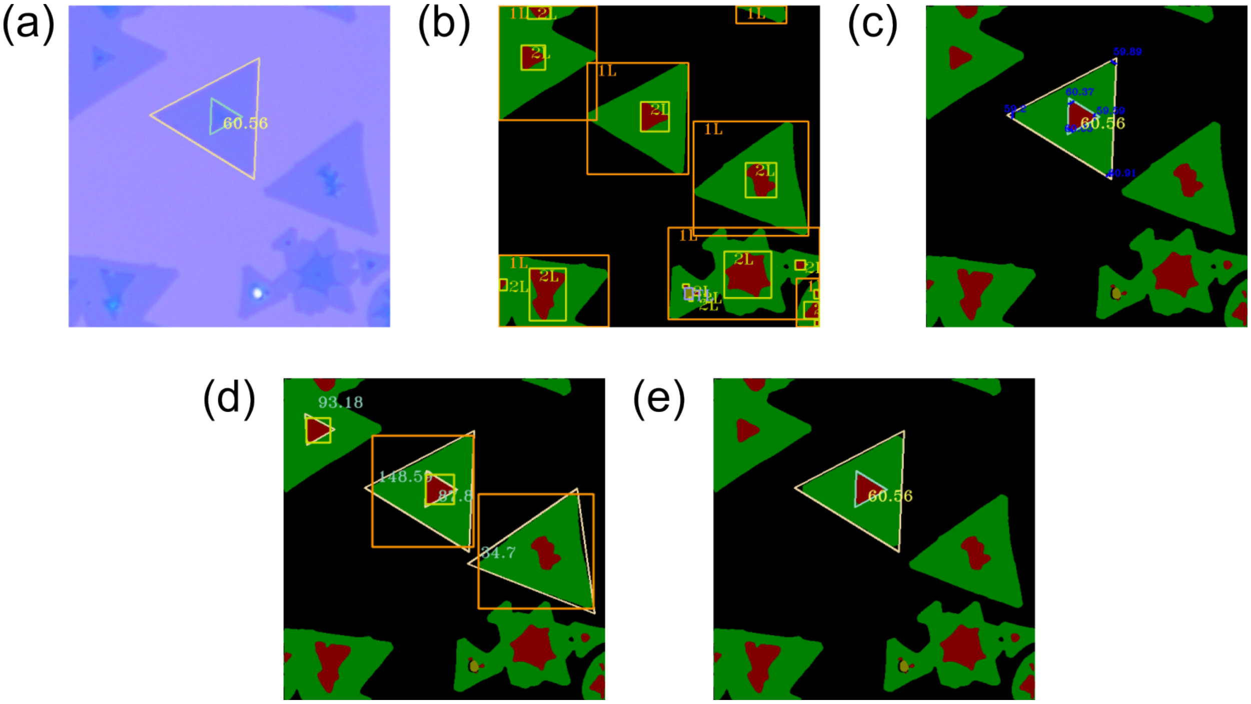

FIG. S12. **Different type of image outputs generated by the image processing module based on different requirements.** The outputs include (a) twist angle information displayed on the original optical microscope image; (b) classification detection performed without the angle information. The corresponding category is highlighted with a label; (c) twist angle information and the interior angle value of the category triangle; (d) all category triangle structures of MoS_2 crystals are identified; (e) the final result image.

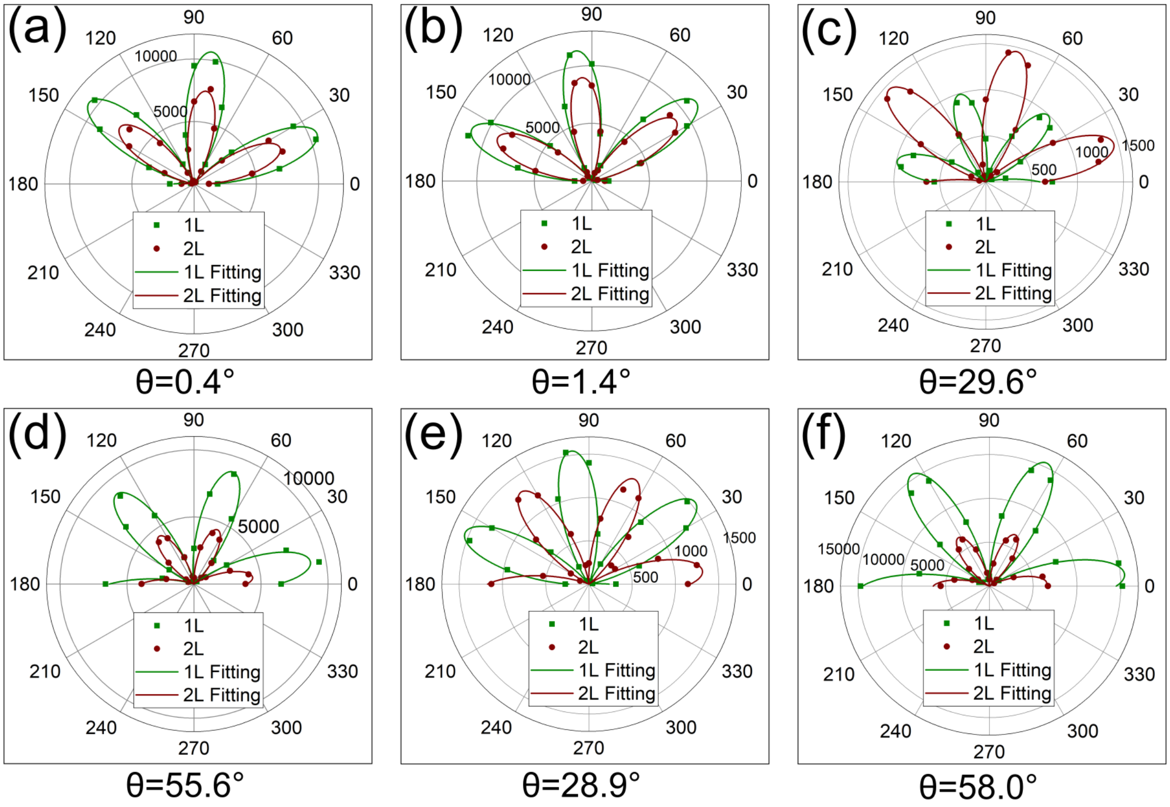

FIG. S13. **Verification of predicted models through SHG analysis of stacked bilayer CVD-grown MoS_2 samples.**

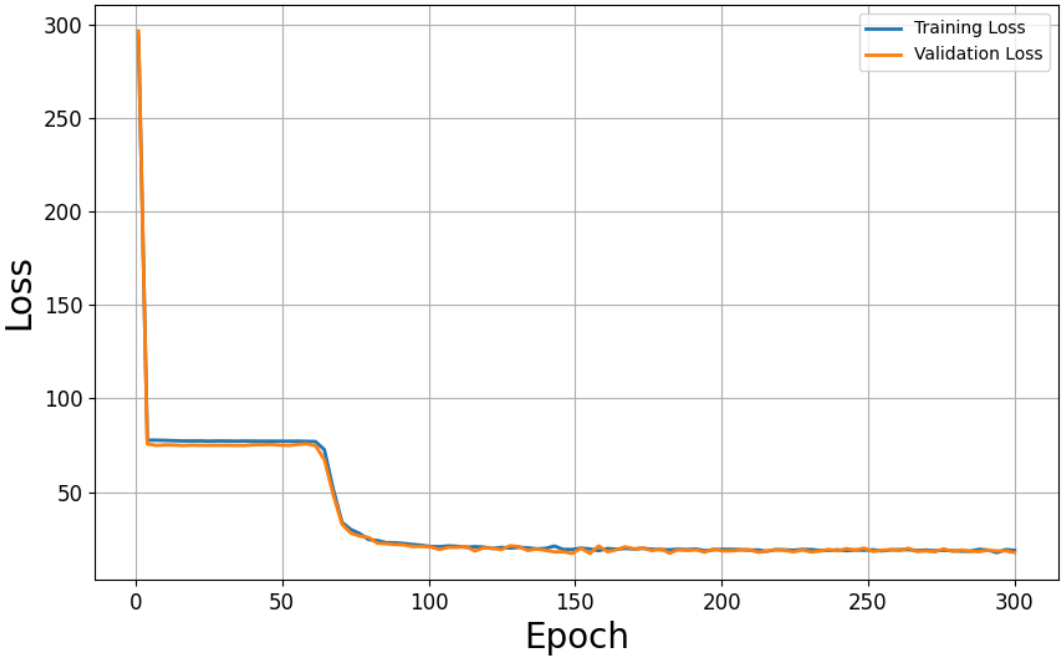

FIG. S14. **Loss function evolution of Graphene bilayer recognition during the training process.**

TABLE S2. **The comparison of the time spent in each step of the recognition process.**

		Thickness Detection Model				*Twist Angle Recognition Model*
Model Name		*DeepLabV3*	*FCN*	*LR-ASPP*	*U-Net*	*ResNet*
Training Epoch		300	300	300	300	600
NN Training time		29m24s	25m18s	17m18s	34m18s	14h25m33s
Frames per second (FPS)	CPU	1.16	1.32	9.80	3.56	14.3
	GPU	45.66	50.35	201.25	125.63	479.6

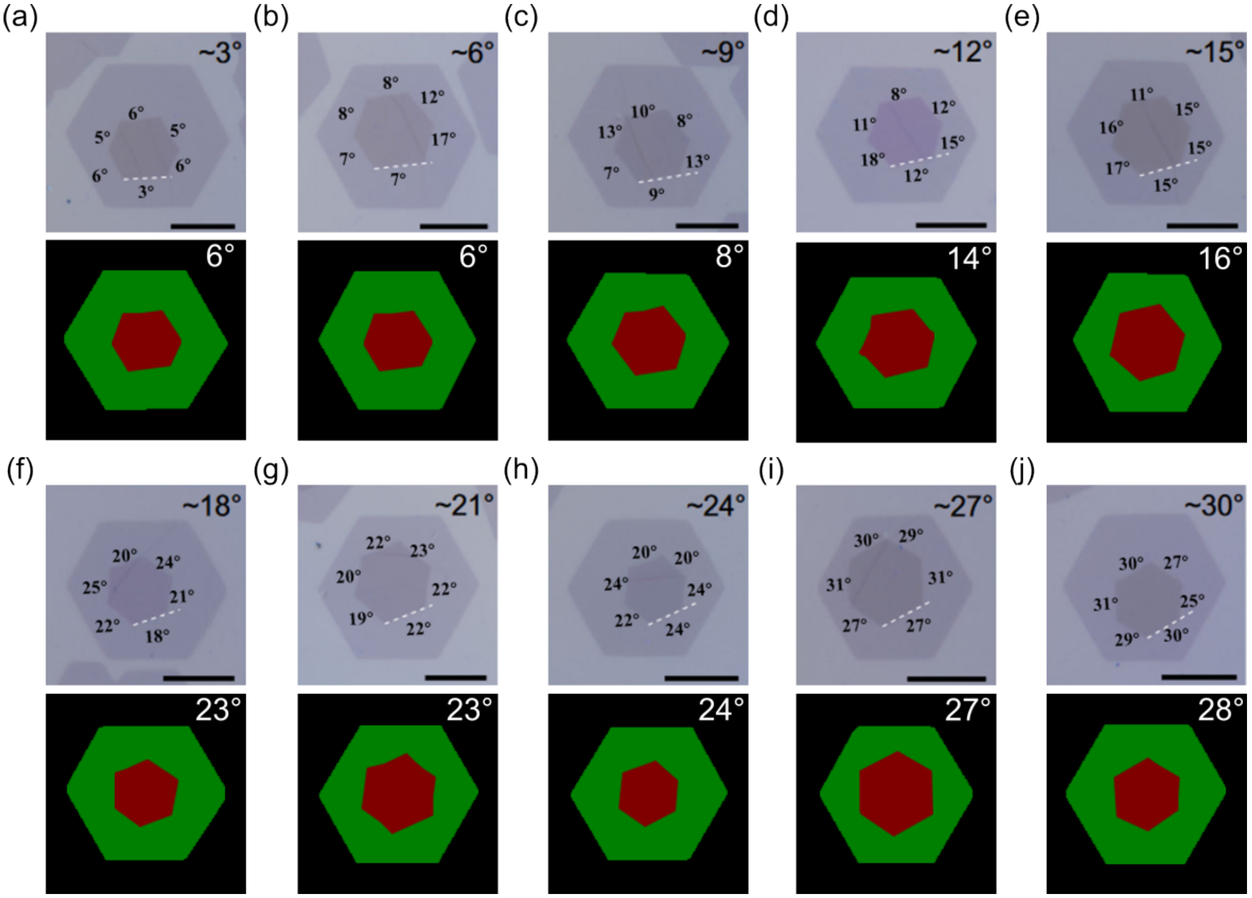

FIG. S15. **Twist angle prediction of the CVD-grown twisted bilayer Graphene, trained by our CNN model, adapted from the work of Sun et al. [11].** The optical microscope photograph at the top, originally inspired by Sun et al.s research [11], displays manually annotated twist angles between the edges of the second and first layers. The corresponding thickness identification photo below is annotated with angles identified by our second CNN model. These adaptations and modifications are made in accordance with the Creative Commons Attribution 4.0 International License (CC BY 4.0), which permits unrestricted use, distribution, and reproduction in any medium, provided the original work is properly cited. For details of the original work, see Sun et al. [11], published by Nature Publishing Group UK London in Nat. Commun., volume 12, number 1, pages 2391, 2021. A link to the license's text: http://creativecommons.org/licenses/by/4.0/. Scale bars: 10 μm.

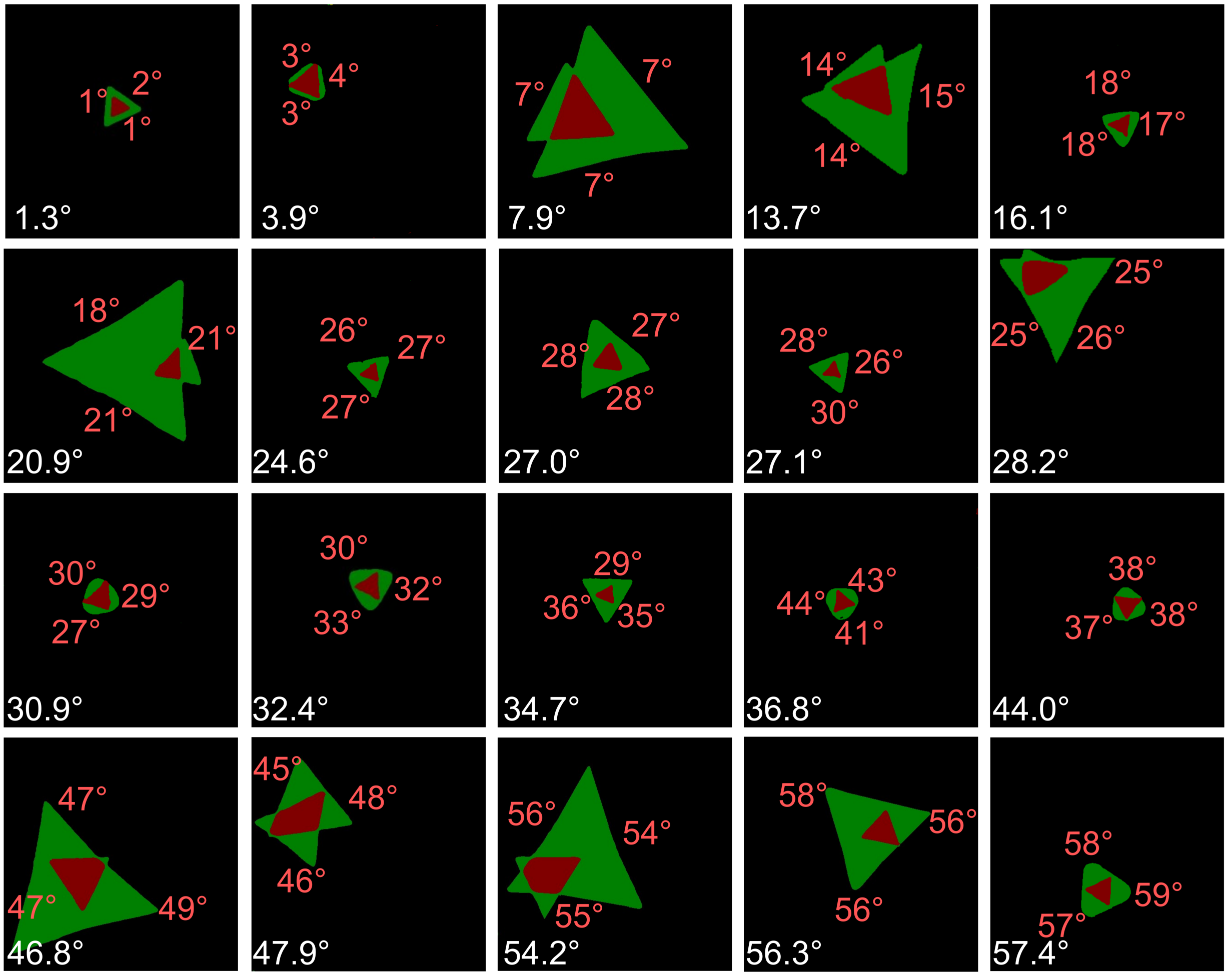

FIG. S16. **The 20 samples of bilayer MoS_2 with varied twist angles as shown in Fig.5.** This figure showcases 20 samples, each illustrating the effectiveness of the CNN-based model in predicting twist angles (indicated in the bottom-left corner of each subfigure), compared to angles obtained via OpenCV (displayed in the bottom-right corner). The red markings highlight the manually annotated twist angles between the edges of the second layer and the first layer of each sample.

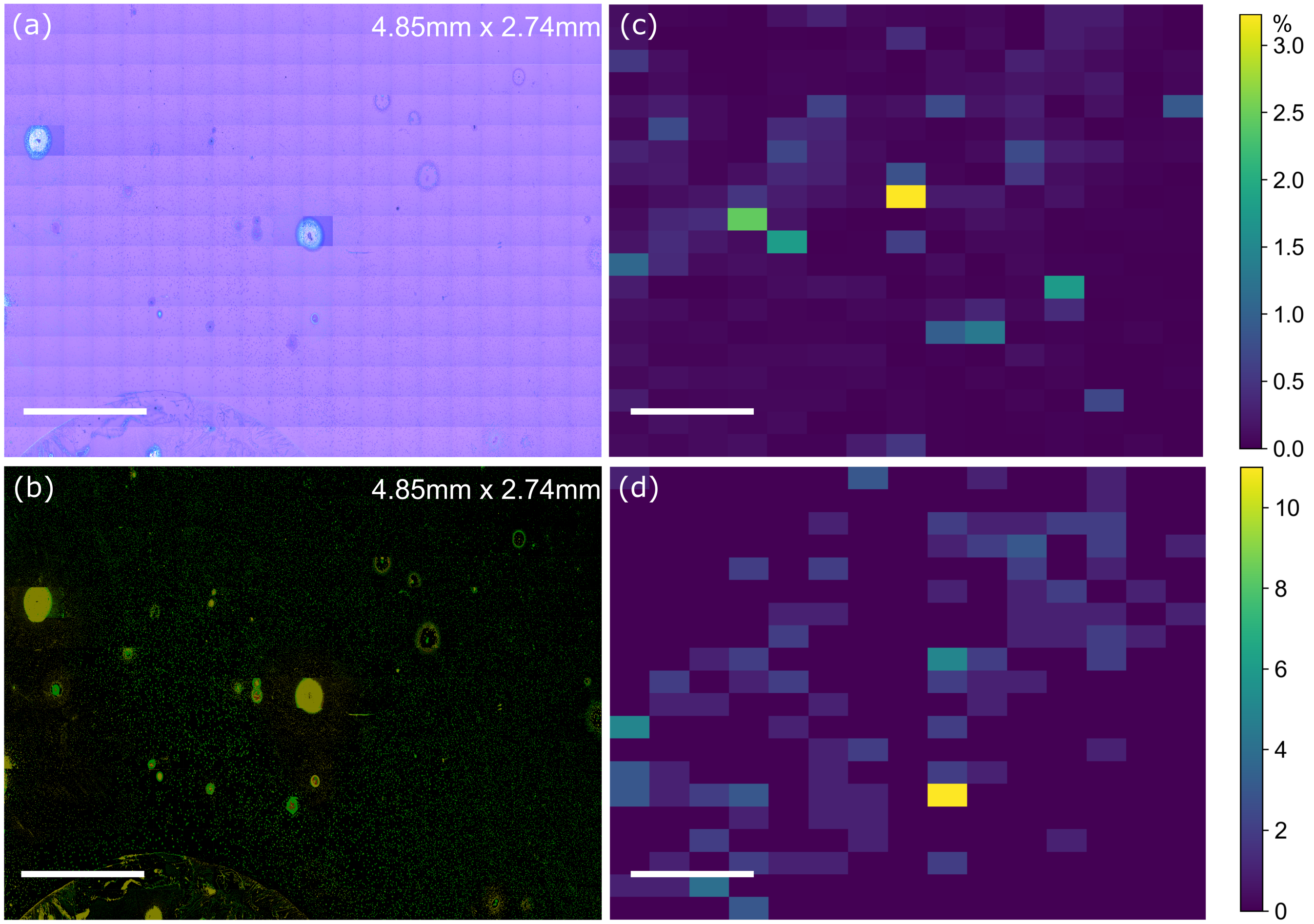

FIG. S17. **The large-scale mapping of the distribution of the twisted bilayer MoS_2.** (a) a large image composed of stitched original 500× photographs; (b) a thickness photo obtained through recognition of (a); (c) represents the area ratio of double layers to the size of a single original optical microscope image; (d) the number of twisted bilayer MoS_2 automatically identified in each original image, corresponding to the original 500× photographs.

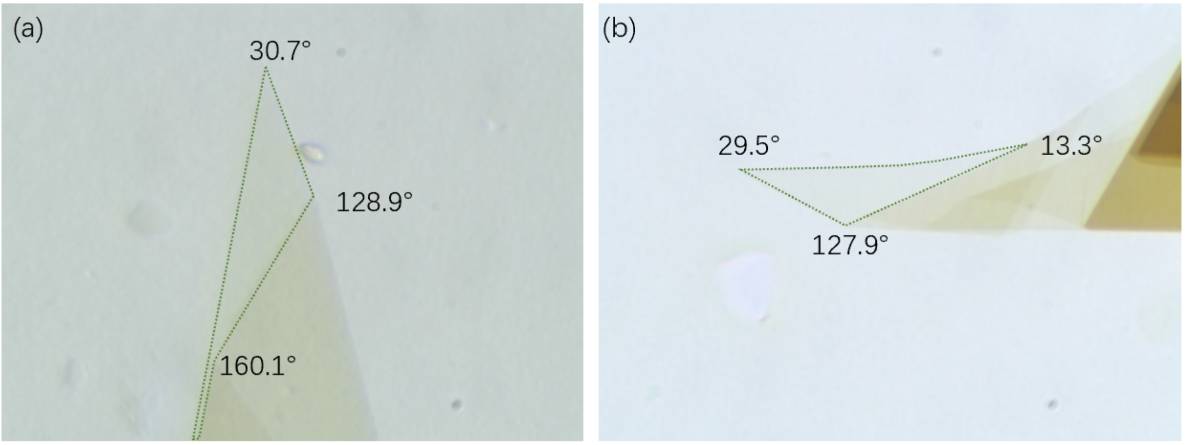

FIG. S18. **The "good" 1L exfoliated MoS_2 flakes used for building the artificial hetero- or homo-moiré structures.**

References

* These authors contributed equally to this work

† yan.zhou@bristol.ac.uk

‡ phtan@semi.ac.cn

§ Eduardo.Hernandez@csic.es

¶ yxie@xidian.edu.cn

[1] Y. Xie, Twist2dnet, `https://github.com/YongXie-ICMM/Twist2DNet.git` (2024), accessed: February 10, 2024.

[2] Y. Xie, Z. Wang, Y. Zhan, P. Zhang, R. Wu, T. Jiang, S. Wu, H. Wang, Y. Zhao, T. Nan, and X. Ma, Controllable growth of monolayer MoS_2 by chemical vapor deposition via close MoO_2 precursor for electrical and optical applications, Nanotechnology **28**, 084001 (2017).

[3] Z. Wang, Y. Xie, H. Wang, R. Wu, T. Nan, Y. Zhan, J. Sun, T. Jiang, Y. Zhao, Y. Lei, M. Yang, W. Wang, Q. Zhu, X. Ma, and Y. Hao, Nacl-assisted one-step growth of MoS_2–WS_2 in-plane heterostructures, Nanotechnology **28**, 325602 (2017).

[4] Y. Xie, X. Ma, Z. Wang, T. Nan, R. Wu, P. Zhang, H. Wang, Y. Wang, Y. Zhan, and Y. Hao, Nacl-assisted cvd synthesis, transfer and persistent photoconductivity properties of two-dimensional transition metal dichalcogenides, MRS Adv. **3**, 365 (2018).

[5] M. Everingham, L. Van Gool, C. K. Williams, J. Winn, and A. Zisserman, The pascal visual object classes (voc) challenge, Int. J. Comput. Vis. **88**, 303 (2010).

[6] D. E. Rumelhart, G. E. Hinton, and R. J. Williams, Learning representations by back-propagating errors, Nature **323**, 533 (1986).

[7] L.-C. Chen, G. Papandreou, F. Schroff, and H. Adam, Rethinking atrous convolution for semantic image segmentation (2017), accessed on December 06, 2023.

[8] J. Long, E. Shelhamer, and T. Darrell, Fully convolutional networks for semantic segmentation, in *Proceedings of the IEEE conference on computer vision and pattern recognition* (2015) pp. 3431–3440.

[9] A. Howard, M. Sandler, G. Chu, L.-C. Chen, B. Chen, M. Tan, W. Wang, Y. Zhu, R. Pang, V. Vasudevan, Q. V. Le, and H. Adam, Searching for mobilenetv3, in *Proceedings of the IEEE/CVF international conference on computer vision* (2019) pp. 1314–1324.

[10] O. Ronneberger, P. Fischer, and T. Brox, U-net: Convolutional networks for biomedical image segmentation, in *Medical Image Computing and Computer-Assisted Intervention–MICCAI 2015: 18th International Conference, Munich, Germany, October 5-9, 2015, Proceedings, Part III 18* (Springer, 2015) pp. 234–241.

[11] L. Sun, Z. Wang, Y. Wang, L. Zhao, Y. Li, B. Chen, S. Huang, S. Zhang, W. Wang, D. Pei, H. Fang, S. Zhong, H. Liu, J. Zhang, and et al., Hetero-site nucleation for growing twisted bilayer graphene with a wide range of twist angles, Nat. Commun. **12**, 2391 (2021).
